# Comprehensive review of the genotype-phenotype spectrum and treatment for biallelic insulin receptoropathy based on 259 affected people

**DOI:** 10.21203/rs.3.rs-11296131/v1

**Published:** 2026-11-01

**Authors:** Sheldon D'Silva, Bruno Donadille, Jingxiang Xu, Christine Bellanné-Chantelot, Vahid Aslanzadeh, Delphine Collin-Chavagnac, Jamal Al Jubeh, Rakesh Amin, Jacques Beltrand, Jean-Paul Beressi, Frédéric Beziaud, Elise Bismuth, Geremia Bolli, Jean-Claude Carel, Kate Chandler, Elaine Cochran, Marc de Kerdanet, Annalisa Deodati, Valeria Grasso, Siri Greeley, Iva Gueorguieva, Tulay Guran, Sekar Kanthimathi, Louis Lebreton, Mariangela Martino, Viswanathan Mohan, Benjamin Moreau, Sze May Ng, Marc Nicolino, Marek Niedziela, Kevin Perge, Michel Polak, Radha Venkatesan, Rachel Reynaud, Megan Startzell, A Kemal Topaloglu, Amaia Vela Desojo, Carine Villanueva, Rachel Williams, Michael Witsch, Khalid Hussain, Anna Stears, David Savage, Mehul Dattani, Loredana Marcovecchio, Stephen O'Rahilly, Camille Vatier, Isabelle Jéru, Kevin Colclough, Fabrizio Barbetti, Corinne Vigouroux, Rebecca Brown, Robert Semple

**Affiliations:** University of Edinburgh; Centre de Recherche Saint-Antoine; University of Edinburgh; Pitié-Salpêtrière Hospital; University of Edinburgh; Hospices Civils de Lyon; Shaikh Khalifa Medical City; Great Ormond Street Hospital; Hôpital Necker-Enfants Malades; Centre Hospitalier de Versailles; Centre Hospitalier de Versailles; Hôpital Robert-Debré; University of Perugia; Hôpital Robert-Debré; Manchester University NHS Foundation Trust; National Institute of Diabetes and Digestive and Kidney Diseases; Centre Hospitalier Universitaire de Rennes; Bambino Gesù Children's Hospital; University of Rome Tor Vergata; University of Chicago; Centre Hospitalier Universitaire de Lille; Marmara University; Madras Diabetes Research Foundation; Hôpital Pellegrin; Bambino Gesù Children's Hospital; Madras Diabetes Research Foundation; Centre Hospitalier Universitaire de Rennes; Edge Hill University; Hospices Civils de Lyon; Poznan University of Medical Sciences; Hospices Civils de Lyon; Hôpital Necker-Enfants Malades; Madras Diabetes Research Foundation; Hôpital de la Timone; National Institute of Diabetes and Digestive and Kidney Diseases; Massachusetts General Hospital; Hospital de Cruces; Hospices Civils de Lyon; Nottingham University Hospitals NHS Trust; Centre Hospitalier de Luxembourg; Sidra Medical and Research Center; Cambridge University Hospitals NHS Foundation Trust; University of Cambridge; Great Ormond Street Hospital; Cambridge University Hospitals NHS Foundation Trust; University of Cambridge; Centre de Recherche Saint-Antoine; Pitié-Salpêtrière Hospital; Royal Devon University Healthcare NHS Foundation Trust; Bambino Gesù Children's Hospital; Sorbonne Université; National Institute of Diabetes and Digestive and Kidney Diseases; University of Edinburgh

**Keywords:** INSR, insulin receptor, insulin resistance, Donohue syndrome, Rabson Mendenhall syndrome, leprechaunism, genotype–phenotype, hypoglycaemia, diabetes, machine learning

## Abstract

**Background:**

Biallelic deleterious variants in the insulin receptor gene (*INSR*) cause severe insulin resistance (IR), historically partitioned into Donohue syndrome, Rabson–Mendenhall syndrome and type A IR. Case literature is scattered, and clinical management rests on uncontrolled case series. Genotype–phenotype relationships, natural history, prognosis and optimal management are only partially established.

**Methods:**

We reviewed reports of biallelic INSR variants published over 40 years and supplemented these with cases from international referral centres, abstracting clinical, biochemical and genetic data from 259 affected individuals (226 unrelated probands; 210 distinct INSR variants). Variant burden was compared with the general population using gnomAD, and a Random Survival Forest-based prognostic model was developed, with cross-validation of feature importance.

**Results:**

Complete loss-of-function (KO) variants affected 33.3% of patients vs 12.6% in gnomAD (p = 2.5×10^−12^), with no domain clustering. All biallelic KO patients presented in the first month of life. Disease was multi-system, encompassing profound IR with fasting hypoglycaemia, hyperglycaemia without dyslipidaemia, pre-/postnatal growth failure with soft-tissue overgrowth, cholestatic liver disease, nephrocalcinosis, cardiac hypertrophy, ovarian enlargement, coagulopathy and colonic overgrowth. Seventy-two individuals (28%) died at a median 0.36 years, most commonly from sepsis; all KO patients were deceased or censored by 36 months. The prognostic model discriminated survival beyond 2 years, stratifying the cohort into tertiles with 2-year survivals of 89.8%, 83.3% and 39.5%. Biallelic KO variants and low birthweight standard deviation score were the strongest negative predictors, with male sex also adverse. Evidence guiding therapy is weak: insulin and metformin remain pragmatic mainstays, SGLT2 inhibitors show encouraging benefit, and rhIGF-1, used in a third of cases, yielded mixed outcomes.

**Conclusions:**

Biallelic insulin receptoropathy forms a clinical continuum. Routinely available presentation features permit data-driven prognostication, facilitating individualised risk assessment. Several stereotyped tissue-specific features remain poorly understood mechanistically. Strengthening of the therapeutic evidence base is urgently required.

## INTRODUCTION

Insulin is essential for human life due to its pre-eminent role in transducing nutritional cues into metabolic responses. It exerts actions on nearly all tissues by binding to a dimeric membrane-associated tyrosine kinase receptor encoded by the *INSR* gene [1]. Human disease-causing variants in *INSR* were identified in human severe insulin resistance (IR) in 1988 [2, 3], swiftly following description of the nucleic acid sequence of the *INSR* gene in 1985 [4, 5]. In ensuing years a rush of mutation discovery led to rapid delineation of the genotype and phenotype spectra of *INSR*-related disorders, and the view summarised in the early 1990s [6, 7] still applies today [8].

Human *INSR*-related severe IR encompasses autosomal recessive insulin receptoropathies caused by pairs of loss-of-function variants in any part of the receptor, and monoallelic IR caused by heterozygous mutations [6, 7]. Variants causing monogenic *INSR*-related IR have most widely been viewed as lying in the intracellular region of the receptor and exerting dominant negative action on co-expressed wild-type half receptors [9-13], however increasing evidence suggests that heterozygosity for simple loss-of-function (LoF) mutations also causes severe IR, often in conjunction with stressors such as puberty (companion monoallelic manuscript already submitted to *Genome Medicine*).

The phenotypic spectrum of *INSR* defects is wide, ranging from neonatal extreme IR, growth retardation *in utero* and beyond, and death in the first year of life, to insulin resistant diabetes or occasionally hypoglycaemia presenting in mid-life. The clinical nomenclature used to capture this spectrum is variably applied. It includes terms such as “leprechaunism”, coined in early clinical descriptions of the severe end of the spectrum [14], and later sometimes used even for adult patients with *INSR* mutations. This term is now largely eschewed due to lack of scientific utility and pejorative connotations in favour of Donohue Syndrome, based on the same index clinical description of the lethal recessive syndrome [14]. Rabson Mendenhall syndrome, also based on early description of a condition with intermediate severity identified in childhood [15], and Type A IR, denoting peri- or post-pubertal severe IR and hyperandrogenism in women [16], complete the spectrum. No settled terminology is used for post-pubertal men with *INSR*-related severe IR. We focus here on biallelic insulin receptoropathy exclusively.

Current knowledge of the optimal treatment of people with recessive *INSR* defects is based on uncontrolled case reports and case series [17]. However, the wide range of genotypes and phenotypes, and the imprecisely delineated correlation between them, pose a particular problem when evaluating such reports. Specifically, uncertainty regarding the natural history of different biallelic variant combinations means that it is usually not possible to ascribe an observed relatively good outcome with certainty to therapy. In some, and perhaps many, cases better outcomes may instead reflect a lesser degree of dysfunction of some variant combinations.

In this study we draw on the largest available number of cases with biallelic *INSR* defects, with two goals: first, to offer the most comprehensive summary of the complex, multi-organ pathology of recessive receptoropathy to date, thereby extending understanding of the role of the *INSR* in normal human development and physiology. Second, to use machine learning to improve prognostic stratification of patients with biallelic *INSR* variants, based on clinical, biochemical and genetic features. This stratification may guide future therapeutic studies.

## METHODS

### Ascertainment, ethics

Ascertainment and ethical approvals are described in detail in (companion monoallelic manuscript already submitted to *Genome Medicine*). In brief, we searched PubMed with terms including “monogenic insulin resistance”, “Type A insulin resistance”, “Donohue Syndrome”, “Rabson Mendenhall Syndrome”, “leprechaunism”, “insulin receptor mutation”, or “*INSR*” and “insulin resistance”, and selected all case reports, case series and trials as well as relevant reviews, before secondary review of cited reports not captured by the primary search. We augmented published probands with unpublished patients with biallelic predicted pathogenic and/or loss-of-function (LoF) *INSR* variants identified through genetic testing at referral centres in Cambridge, Paris, the NIH, Exeter, Rome, or Chennai. Reports were grouped into three eras demarcated by therapy licensing landmarks: before 1999, 2000–2012, and 2013 to present, as described in (companion monoallelic manuscript already submitted to *Genome Medicine*).

### Data curation/cleaning

Data curation and cleaning methods are described in (companion monoallelic manuscript already submitted to *Genome Medicine*). In brief, amino acid numbering followed the *INSR* MANE transcript NM_000208.4 (B isoform). Population frequencies were from gnomAD [18] (accessed July 2026). NMD probability was assessed using NMDetective [19], splice-site variants using SpliceAI [20], and variant deleteriousness using CADD v1.7 [21, 22]. As CADD is known to under-score indels [23], the artefactually low CADD score for the c.591delC frameshift variant was replaced with 30. Variants were classified as high-confidence complete LoF if they were: (1) nonsense or frameshift variants predicted to undergo NMD or lying in the EC domain; (2) essential splice-site variants without predicted alternative splice site unmasking by SpliceAI; or (3) deletions of one or more EC exons causing frameshift. EC missense variants were additionally annotated with results from a massively multiplexed assay of variant effect [24]. Patients with biallelic confident complete LoF variants were annotated as functional *INSR* knockouts (KO).

Allele-specific CADD scores were summed for biallelic combinations to generate a combined CADD score. For unscorable variants meeting LoF criteria, the highest observed CADD score (52.0) was assigned. We derived a unified experimental functional score from our recently reported MAVEs [24], using the composite sum of cell surface expression, insulin binding, and maximal signalling. Gaps in the EC missense score matrix were imputed using MAVE-Polish (https://mavepolish.onrender.com/); variants robustly predicted to cause LoF received the lowest experimental score (−3.1); and IC missense variants (at or beyond residue 974) received − 4.6 to account for dominant negative effects [24]. Scores for each allele were summed to generate a unified function score per biallelic combination.

Birthweight SDS were computed using INTERGROWTH-21st [25] and BMI SDS using CDC reference data [26], without accounting for ethnicity. Organ-specific clinical features and treatment use were recorded dichotomously, and longitudinal biochemical measures as single values. Full genetic and clinical data are aggregated in **Additional File 1**.

### Analysis and statistical methods

In variant distribution analyses, individuals were the counting unit. For IC/EC distribution and mutation type frequencies, the denominator was all 259 probands; for gnomAD comparisons, only probands with CADD-scoreable or imputable variants. A CADD PHRED > = 16 threshold was applied to gnomAD variants. IC (407 aa) vs EC (975 aa) variant density ratios were compared using a two-sample Poisson rate ratio test (conditional exact binomial, Clopper-Pearson 95% CI) as described previously (companion monoallelic manuscript already submitted to *Genome Medicine*). Variant-type proportions and IC:EC ratio-of-ratios between cohort and gnomAD were compared using Fisher's exact test with Bonferroni correction; sensitivity analysis across CADD thresholds 15–30.

Categorical comparisons used Fisher's exact test, continuous variables Mann-Whitney test, SDS comparisons the one sample Wilcoxon signed rank test, and time-to-mortality Mantel-Cox log-rank testing, using GraphPad Prism 11.0.0.

### Random Survival Forest Analysis

A clinical prognostic model was built from quality-controlled, collated data. Expanded details of methods are in **Additional File 2**. More briefly, censoring time was age at last contact; survival time was time from presentation to death or censoring. Candidate predictors, selected for clinical plausibility and data completeness, included summed CADD scores and the unified variant function score. Imputation was used to avoid selection bias: probabilistic recoding mapped a feature-specific proportion of "unknown" values to "No" according to clinical expert priors that unreported findings likely indicate true absence, then remaining unknowns were stochastically imputed from within-fold observed distributions. Continuous missing values were median-imputed within each training partition and categorical predictors one-hot encoded with infrequent-category handling; predictors with > 50% missingness were excluded.

Continuous candidate predictors were gestational age, birthweight SDS, summed CADD scores, and summed experimental function scores [24]. Categorical predictors included reporting era, sex, mutation group, diabetes, cardiac/liver abnormalities, coagulopathy, nephromegaly, hypoglycaemia, nephrocalcinosis, and hypothyroidism. Volatile biochemical indices often documented under non-standardised conditions such as plasma insulin concentrations were omitted. Neurocognitive impairment and frequent infections were excluded to avoid survival bias, as these require significant follow up to manifest, meaning their inclusion as baseline predictors would artificially inflate survival estimates.

A Random Survival Forest was trained by dividing the group into five subgroups or “folds”. This enabled cross-validation, with each fold withheld in turn as an independent test set. Calibration was verified by comparing mean predicted versus Kaplan–Meier-observed 2-year survival across the five subgroups, using pooled out-of-fold predictions to prevent optimistic bias. Patients were stratified into Low-, Medium-, and High-risk tertiles. Discrimination was assessed by Harrell's and Uno's C-index, 2-year accuracy by time-dependent AUC and Brier score, and calibration by the Integrated Brier Score (0.1-2.0 years) and predicted versus Kaplan-Meier-observed survival across subgroups. Feature importance was estimated by permutation (30 repeats per fold). Analyses used Python (pandas, NumPy, scikit-survival, scikit-learn, lifelines, matplotlib, seaborn).

### Large Language Model Use

Large Language Models were used only for verifying internal consistency of data presented, and to aid text editing to reduce word count.

## RESULTS

### General characteristics of population studied

Data from 259 affected people were accessible, including 226 probands and 33 relatives. There was a rise in rate of ascertainment since roll out of next generation sequencing from 2009 (Figure 1A). There was an equal distribution of males (n=117) and females (n=129), with 13 people of undocumented sex. Sixty-four patients were reported from UK centres, 40 from the US, 26 from France, 21 from Japan, 15 from Italy, 13 from Saudi Arabia, 11 from China, and 10 or fewer from other countries. Ethnicity was inconsistently documented. Most patients presented in the neonatal period or infancy, however, there was a tail extending to 30 years. Median [25^th^,75^th^ centile] age at last follow up was in infancy for patients with biallelic complete LoF variants (designated “KO”) (0.30 [0.17,0.33], maximum 1.25 years) and ranged from infancy through late adulthood for non-KO patients (9.5 [1.2,17.0], maximum 62 years).

### Genetic architecture of biallelic insulin receptoropathy

210 different *INSR* variants were identified among 226 unrelated probands, 118 homozygous and 108 compound heterozygous (Figure 1B). Counting single probands from independent families only, the most common variants were S350L (16 occurrences in 9 probands (e.g. [27-32])); P220L (14 occurrences in 7 probands); I146M (11 occurrences in 6 probands, all with ancestry traced to Somalia or Yemen (e.g. [33-35])); T937M (9 occurrences in 7 probands [36-40]); and R141W (6 occurrences in 3 probands, all in the Gulf states [41, 42]). 158 variants were seen in only a single proband. Including non-unique variants, there were 297 missense, 54 nonsense, 26 frameshift, 25 essential splice-site and 6 other splice-site variants, 11 in-frame deletions, 25 large deletions or duplications, 4 indels, 1 insertion and 1 lost stop codon (Figure 2A).

The biallelic cohort was significantly enriched for complete LoF variants relative to gnomAD (33.3% vs 12.6%, OR = 3.47, p = 2.5x10^−12^), unlike in monoallelic *INSR*-related IR, where LoF variants were depleted (companion monoallelic manuscript already submitted to *Genome Medicine*). Also strikingly unlike monoallelic disease, which features 14-fold enrichment in IC missense variants, neither missense nor LoF variants in biallelic disease were differentially distributed between IC and EC regions: domain-length-normalised IC:EC density ratios (DR) showed no significant difference: for missense variants, cohort DR was 0.85 (95% CI 0.56-1.26) vs gnomAD 1.14 (1.01-1.27); for LoF variants, cohort DR was 1.25 (0.72-2.12) vs gnomAD 0.86 (0.61-1.18). Both remained non-significant across all CADD thresholds. This proportionate distribution is consistent with absence of a position-specific dominant-negative mechanism, unlike monoallelic disease.

Homozygous variants allow *in vivo* comparison of variant deleteriousness unclouded by uncertainty inherent in functional predictions or limited *in vitro* functional assays. We thus plotted birthweight SDS (a robust and stable readout), against age at last assessment (the most clinically important endpoint) for all homozygous LoF and missense variants or in-frame indels (Figure 2B,C). Homozygous LoF variants were generally associated with very low birthweight SDS and early death. Three splice-site variants were exceptions; two were associated with higher birthweight and prolonged survival, and one with a very low birthweight SDS but surprisingly long survival. One of these variants affects a non-essential splice site, with some residual normal splicing predicted, while one of the essential splice-site variants is predicted to induce an in-frame exon deletion. These variants can thus plausibly be regarded as hypomorphic rather than true LoF. The third variant is confidently predicted to confer complete LoF, but, notably, that patient was treated with recombinant human IGF-1 (rhIGF-1) from the age of 42 months (**Additional File 1**). Missense variants and in-frame indels showed a much wider range of birthweights and survival; around 40% had birthweight SDS above −2.5, early demise was rare, and survival beyond 10 years was common, with most individuals still alive when last evaluated. Most recurring variants (P220L, I146M, R141W, S350L) fell in this group.

### Presenting features

All individuals with functional *INSR* KO presented in the first month of life, while median age at presentation among individuals with other allele combinations was ~3 months (Figure 3A). Median age at last follow up (whether to death or last review) among KO individuals was less than 4 months (range: neonatal to almost 3 years), compared to 10.0 years (range: neonatal to 60 years) in non-KO individuals (Figure 3B). Presenting features are shown in Table 1. In the entire cohort, the most common presenting features were growth failure (44.3%), dysmorphic features (34.7%), hyperglycaemia (30.6%), acanthosis nigricans or laboratory evidence of hyperinsulinaemia (22.8%), signs or symptoms of hyperandrogenism (13.1% of females), and hypoglycaemia (12.3%). KO patients were more likely than others to present with growth failure (81 vs 40.4%, p = 0.0004) and/or dysmorphic features (67 vs 32%, p=0.0028), with no difference in percentages reported with hyperglycaemia, hypoglycaemia, hyperinsulinaemia, or hyperandrogenism at presentation.

### Metabolic phenotype

Frequencies of clinical features observed are reported in Table 2. Diabetes or hyperglycaemia was documented in >90% of individuals; this usually presented in the first decade of life, but onset ranged from birth to adulthood. Hypoglycaemia was described in 103 individuals (72%) and was frequently neonatal, with blood glucose nadirs as low as 0.7 mmol/L. It was predominantly fasting, though postprandial/postabsorptive hypoglycaemia was also described. It was asymptomatic in some cases despite very low glucose concentrations (1.1–1.6 mmol/L), consistent with chronic adaptation. Several patients showed progression from severe infantile hypoglycaemia towards diabetes. Many exhibited alternating hypo- and hyperglycaemia, or hypoglycaemia observed only after insulin or rhIGF-1 initiation.

In longer-term survivors, diabetic microvascular complications occurred at young ages (Table 2). Eye complications were documented in 20 instances, with proliferative diabetic retinopathy (PDR) most common (n=7); blindness occurred in 5 patients (12-35 years). One patient developed PDR precipitated by sharp HbA1c improvement on rhIGF-1 [43]. Renal complications were documented in 30 instances, ranging from microalbuminuria to end-stage renal failure (at 24 and 39 years), appearing from 13.5 years. Neuropathy was rarely reported.

Diabetic ketoacidosis (DKA) was documented in only 16 cases (Table 2), with first episodes from 6 months [44] to 18 years, despite near-complete *INSR* loss in many. DKA recurred in at least 3 patients. Infection was a common trigger. Insulin requirements during DKA were extreme, up to 50,000 units/day [45]. DKA also occurred in single individuals during growth hormone therapy [46] or SGLT2 inhibitor (SGLT2i) treatment (unpublished).

Median fasting insulin (Figure 3C) was extremely high (1729 [712, 3575] pmol/L) with normal median fasting glucose (4.7 [3.0, 9.3] mmol/L), although individual values ranged from hypoglycaemia to severe hyperglycaemia (Figure 3D). Fasting insulin was higher in KO patients than others, despite lower fasting glucose. Non-fasting or post challenge insulin was likewise strikingly elevated (3972 [1860, 6368] pmol/L, Figure 3E), with concomitantly elevated glucose (13.6 [8.7, 19.4] mmol/L, Figure 3F); there were no differences between KO individuals and others, though documentation of differences between fasting and non-fasting values was sparse in many severely affected infants. Median HbA1c was elevated (77 [48, 107] mmol/mol) and did not differ by genotype; however, only 2 KO patients had HbA1c reported (Figure 3G).

Lipid abnormalities, including dyslipidaemia and Metabolic Dysfunction-Associated Steatotic Liver Disease (MASLD) (Table 2) were extremely rare, sharply at odds with monogenic lipodystrophic forms of severe IR [47]. Median serum triglyceride concentration was 0.9 [0.7, 1.2] mmol/L; only 5 patients (14.7%) had triglycerides >1.7 mmol/L, with the highest 5.9 mmol/L, documented transiently on days 3-4 of life [48]. Median HDL-cholesterol (HDL-C) was 1.6 [1.3, 1.9] mmol/L; only 4 patients (12.9%) had HDL-C <1.0 mmol/L, and two of these also had mild hypertriglyceridaemia (**Additional File 3**). Increased serum LDL-cholesterol was noted in 3 patients.

### Growth

Birthweight SDS (Figure 3H) was available in 136 patients (52%). Median [25^th^, 75^th^ centile] birthweight was −2.8 [−3.3, −1.8], significantly lower than the population average (p<0.0001). Birthweight SDS was lower in KO patients than non-KO patients (−3.6 [−4.2, −2.9] vs −2.7 [−3.3, −1.7]; p=0.0002). Impaired postnatal growth (Table 2) was present in all KO patients, with postnatal weights usually 5-7 SD below the mean. 74.3% of non-KO patients also showed reduced postnatal growth, though a wide range of height outcomes was seen. Many documented adult heights fell 2-4 SD below normal, although a minority reached the normal range.

Skeletal abnormalities included brachydactyly, scoliosis, rhizomelic limb shortening, rickets, and osteoporosis. At odds with linear growth impairment, a variety of features reminiscent of excess GH/IGF1 action were also seen, including prognathism, macroglossia, and other features of pseudoacromegaly. Body composition was also commonly reported as abnormal. Reduced adipose tissue and/or muscle mass were commented on in the majority (71%) of patients, including all but one KO patient, but in those surviving to older ages a range of different patterns of body composition were seen. Several reports describe relatively preserved or frankly increased adiposity in the lower limbs ([49, 50], follow up of patient IV-7 from [34], two further unpublished cases) (**Additional File 1**).

### Organ/system-specific phenotypic features

#### Reproductive:

Hyperplasia of sex-hormone dependent tissues including genitalia and nipples was noted in 73% of the cohort, including all KO patients (Table 2). A wide range of age at pubertal onset and trajectories were reported. Four individuals reportedly showed precocious puberty, in two of whom breast development was observed at 3-4 years old, despite prepubertal hypothalamic-pituitary-gonadal activity. Frankly delayed puberty and/or hypogonadotrophic hypogonadism was reported in 4 individuals, and delayed menarche or primary amenorrhoea after normal onset of puberty in 11. Among 8 individuals in whom age at menarche was reported, the median age was delayed at 15.5 [14.3, 16.0] years. Postpubertally, severe hirsutism was frequent, sometimes sufficient to warrant ovariectomy.

Enlarged, polycystic ovaries were reported in 90% of females (Table 2), often detectable neonatally. Two patients had ovarian tumours: one a 1.2 kg juvenile granulosa cell tumour (JGCT) with desmoplastic implants after rhIGF-1 [51], another bilateral JGCT treated by ovariectomy at 7 weeks, leading to postoperative death [52-54]. Mechanical complications from enlarged ovaries included respiratory compromise [55] and colonic compression [56]. Ovarian torsion occurred in 2 cases. Oophorectomy was undertaken in at least 7 cases. Tumours also occurred in oestrogen-responsive tissues: two endometrial cancers (at 24 and 28 years old) and one breast cancer at 24 years [57]. In males, the only reproductive complication beyond infantile macrogenitalia was a testicular seminoma at 23 years [57].

#### Renal:

Nephromegaly was reported in 60% of patients (Table 2), and nephrocalcinosis in 75% (Table 2), with no difference in either by genotype. Nephrocalcinosis was reported antenatally in at least 1 case [58], and neonatally in others (e.g. [59]), and yellow papillary streaks and/or calcified intratubular depots were reported at autopsy (e.g. [60, 61]). Hypercalciuria was documented in several cases and a Bartter syndrome-like phenotype was reported in 3 patients [62-64]. One patient was described as having a partial Fanconi syndrome [59], and another had renal tubular dysfunction with sodium, potassium, calcium, magnesium and chloride wasting, leading to hyponatraemia, hypokalaemia and alkalosis at 12 weeks of age [65]. Four further patients had hypokalaemia, one also with hyponatraemia. Polycystic kidneys were reported in 2 patients, and one patient had a congenital solitary kidney.

#### Cardiovascular:

Cardiac abnormalities were reported in 71% of patients (Table 2). Most had ventricular or septal hypertrophy (n=28), with 13 having hypertrophic obstructive cardiomyopathy, usually diagnosed neonatally and occasionally antenatally [65, 66]. Four cases showed worsening hypertrophy on rhIGF-1 therapy, while two with pre-existing severe hypertrophy improved on continuous rhIGF-1 infusion [65, 67]. In some cases hypertrophy improved or resolved spontaneously after infancy. Atrial septal defects (9), ventricular septal defects (2), patent ductus arteriosus (4), and patent foramen ovale (1) were also reported. Death was ascribed to heart failure alone in 2 patients and was a reported cofactor with infection in many more.

#### Hepatobiliary:

Liver disease was reported in 56 patients (55%), more prevalent in KO than non-KO (89 vs 52%, p=0.04, Table 2). A cholestatic pattern was most common (29 individuals), with bilirubin as high as 262 mmol/L. One liver was described as 'almost black' at laparotomy at 7 weeks [68] and others as green at autopsy. Autopsy findings included cholestasis with bile duct casts [60], canalicular cholestasis with portal/periportal fibrosis [65], cholangiolar proliferation (follow up from [69]), bile thrombi in pericentral sinusoids [70], and hepatitis [71]. Hepatomegaly was reported in 12 individuals. Liver failure was not a reported cause of death, but coagulopathy occurred in 12 individuals, sometimes vitamin K-responsive. One infant died from disseminated intravascular coagulation following gastrointestinal haemorrhage [72].

#### Gastrointestinal:

Abdominal protuberance was reported in 77%, more frequent in KO individuals (100 vs 73%, p=0.04, Table 2). Colonic hypertrophy and/or rectal prolapse occurred in 12 patients, including two with diffuse colonic polyposis and benign histology [73], [33], and one who had a single polyp (follow up from [55]). Three had colonic dilation [74, 75], one also with pancreatic exocrine insufficiency [74].

#### Neurocognitive:

Neurocognitive impairment was reported in 31% of patients, with no difference by genotype group (Table 2). It was most commonly described as global developmental delay, delayed motor milestones, or sometimes speech and language impairment. Patients with a history of hypoglycaemia were more likely to have neurocognitive impairment than those without hypoglycaemia (67% vs 26%, p=0.001). Impairment was of variable severity, ranging from mild delay to marked delay and moderate intellectual disability. Cortical atrophy was reported in one individual at 5 years after prolonged neonatal hypoglycaemia. Despite the prominence of pineal hypertrophy in early descriptions of Rabson Mendenhall syndrome, the only pineal abnormality reported was a cyst in a single patient [76].

#### Skin:

Acanthosis nigricans and/or skin tags, and hypertrichosis were reported in 87% and 92% of patients, respectively, with no difference between KO and other genotypes. Skin infections, including candidiasis and hidradenitis suppurativa, were reported in 9 patients, with ill-defined, recurrent psoriasiform lesions in 2 of them [64, 77].

#### Infections:

Frequent infections were reported in 31 individuals (72%), with no difference by genotype (Table 2). Those with frequent infections had higher HbA1c compared to those who did not (98 [80, 131] vs 50 [34, 80], p=0.007). Acute otitis media was the most frequent single infection, often recurrent and requiring tympanostomy tubes, with skin infections also common. Invasive and life-threatening infections with organisms including Group B *Streptococcus*, *Staphylococcus epidermidis*, and *Enterococcus*, endocarditis, osteomyelitis, soft tissue abscesses, *E. coli* pyelonephritis, and *Pneumocystis jirovecii* pneumonia were also reported.

#### Otorhinolaryngological:

Frequent otitis, including otitis externa and otitis media, was reported in 11 patients; in 3 patients this was complicated by mastoiditis and/or cholesteatoma. Tonsillar and/or adenoidal hypertrophy were explicitly documented in 25 cases (Table 2). Tonsillectomy and/or adenoidectomy was reported in 9 cases, with 4 procedures in patients on rhIGF-1 therapy [36, 39, 78, 79], in most cases necessitated by obstructive sleep apnoea. Two patients required tracheostomy.

#### Maxillofacial:

Facial dysmorphism, most commonly described as acromegaloid, elfin, or both at different stages, was reported in 84% of patients, with no difference between KO and other genotypes (Table 2). Gingival hyperplasia was reported in 73% of cases, again with no difference between KO and other genotypes (Table 2). Dental abnormalities were reported in 87% of individuals (Table 2). Severe dental crowding was most common, frequently accompanied by malocclusion, macrodontia and caries, sometimes requiring multiple extractions. Advanced dental maturation was a striking and recurrent feature, encompassing precocious primary and adult dentition, often discordant with retarded bone age. Enamel hypoplasia and poorly calcified canines were reported in several cases.

#### Thyroid:

Central hypothyroidism was reported in 5 individuals [59, 66, 67, 80, 81], with onset as early as 2 months. Goitre, multinodular goitre, and thyroid nodules were documented in older patients. Three patients had papillary thyroid cancer (PTC), one a male patient at 18 years old who also had a testicular seminoma [57], one a female patient, treated with rhIGF-1 since infancy, who developed PTC at 15 years old (follow-up of [55]), and one a female patient who had metastatic PTC at 32 years old, and who also had breast cancer [57].

### Longevity and causes of death

Seventy-two patients (28%) died, at median age 0.36 [0.25 - 1.36] years. Causes of death (39 reported) included infection or sepsis (64%) and cardiorespiratory failure (41%). Deaths were more frequent in KO than non-KO individuals (73 vs 24%, p<0.0001). Kaplan-Meier analysis (Figure 4A) showed all KO patients deceased or censored by 36 months (p<0.0001). Inspection of other mutational subgroups revealed no obvious differences in outcome, though an approximately similar subset of individuals in each group died in the first 2 years, as for the KO patients. Death before 2 years has been regarded as defining Donohue syndrome; these observations support this as a clear subgroup. However, identifying such patients prospectively among non-KO groups remains challenging, motivating the prognostic model below.

### Random survival forest analysis

Given limitations of prognostication, we set out to evaluate predictors of outcome at presentation using Random Survival Forest (RSF) machine learning, an approach that makes no prior assumptions about which variables matter or how they relate to outcome. The primary outcome evaluated was the time from presentation to death or censoring, which was taken as survival time. Candidate prognostic variables were selected based on clinical plausibility, data completeness, and robustness of the reported phenotype or measure. A categorical imputation strategy filled in undocumented categorical phenotypes, drawing on clinical judgement of the meaning of lack of documentation, as detailed in **Additional File 2.**

The RSF model was applied to 155 individuals, among whom 51 died, while 104 were censored during follow up, indicating a well-balanced sample for forecasting. A cross-validation protocol was used wherein the dataset was split into five distinct subgroups, with each group maintaining the same proportion of deaths. Each group was withheld in turn to serve as an independent test set, while the remaining groups were pooled as the training set. For each independent test set, the RSF model generated a prognostic risk score, with higher scores indicating worse predicted outcomes. A target prognostic horizon was set at 2.0 years following presentation, given preliminary survival analysis (Figure 4A) and the suggested survival cut off of 2 years as a discriminator of Donohue Syndrome.

The RSF model demonstrated high accuracy in discriminating clinical trajectories. The pooled out-of-fold Harrell’s concordance index was 0.79, while in cross-validation, the model demonstrated solid prognostic stability, yielding a mean Harrell’s C-index of 0.80 (SD = 0.08; 0.68 - 0.90 across folds). When accounting for censoring patterns during follow up, the mean Uno's C-index remained strong at 0.72 (SD = 0.16). Short-term prognostic accuracy at 2 years post-presentation was evaluated using a dynamic Area Under the Curve (AUC), yielding a mean value of 0.84 (SD = 0.11) and a maximum of 0.97 across folds, indicating reliable capacity to predict absolute mortality during the early post-presentation phase. Overall calibration was excellent, with a mean 2-year Brier score of 0.14 (SD = 0.01; range 0.13-0.15). Further performance metrics, their meaning and interpretation are summarised in **Additional File 2**.

Dividing prognostic risk scores into tertiles segregated the cohort into three distinct clinical survival profiles (Figure 4B,C). The Low-risk group (n = 52; mean baseline risk score 2.78) experienced only 6 deaths, maintaining an observed Kaplan-Meier 2-year survival probability of 89.8%. All 3 of these 6 low risk individuals for whom causes of death were documented died of fulminant infections. The Medium-risk cohort (n = 51; mean baseline risk score 6.88) experienced 11 deaths, giving a 2-year survival probability of 83.3%. In contrast, the High-risk cohort (n = 52; mean baseline risk score 15.11) demonstrated poor prognosis, with 34 deaths and a 2-year survival probability of only 39.5%.

Shuffling input variables identified key drivers of mortality among the candidate predictors (Figure 4D). KO versus non-KO status was the single most powerful predictor, in keeping with prior Kaplan Meier analysis (Figure 4A), with a mean permutation importance score of 0.049 (SD 0.034). This was followed by birthweight SDS (0.032 (SD 0.026), early-onset coagulopathy (0.026 (SD 0.021)), sum of CADD scores (0.01 (SD 0.011)), sex (0.013 (SD 0.012)), and liver pathology (0.012 (SD 0.005)), and the reporting era (0.011 (SD 0.01)). The experimentally determined Unified Function Score, baseline hypoglycaemia, and thyroid dysfunction provided negligible or negative predictive value.

In view of several severe documented ovarian complications, we expected that the sex disadvantage would lie with females, however the opposite proved true: sex-based out-of-fold survival analysis showed male infants to have more than double the raw mortality rate of females (45.3% [34/75] vs 21.3% [17/80]). Moreover, the low-risk group from the RSF was heavily female-skewed (44 females vs 8 males; 85% female), with the high-risk group reciprocally male-dominated (36 males vs 16 females; 69% male).

### Treatment

Among 259 patients, 142 had reports of medication use for diabetes, IR, and/or hypoglycaemia. For the 117 patients with no reported medication use, it was not possible to determine if medications were not used or not reported. Among the 142 with known medication use, 44% used a single drug, 25% used two drugs, 15% used three drugs, 9% used 4 drugs, and 7% used 5 or more drugs. The most commonly reported drug classes were insulin (N=92; 65%), metformin (N=82; 58%), rhIGF-1 (N=49; 34.5%), SGLT2 inhibitors (SGLT2i; N=18; 13%), and thiazolidinediones (N=17; 12%). Fewer than 10% used incretin agonists, metreleptin, or other classes. 5% of patients received medical or surgical therapy for hyperinsulinaemic hypoglycaemia, including diazoxide (5 cases (e.g. [34, 82]; one later undergoing subtotal pancreatectomy on two occasions [34]) and octreotide (1 case [72]).

Medication use followed expected temporal patterns (Figure 5), with SGLT2i use increasing since 2013 (17% in that period). Insulin doses ranged from high (1.5 units/kg/day) to extreme (>750 units/kg/day; maximum 48,000 units/day). Dietary measures, including continuous enteral feeding for fasting hypoglycaemia and carbohydrate restriction for postprandial hyperglycaemia, were documented in 39 individuals (25%).

Most classes of common anti-diabetic drugs had little or no rigorous documentation of efficacy, with the limited evidence presented often confounded by combination therapy. The most promising preliminary evidence relates to SGLT2i, whose mechanism of glucose lowering is *INSR*-independent. SGLT2i use was reported in 18 individuals. Among 12 cases where comment on efficacy was offered, glycaemic benefit was reported in 10 (e.g. [44, 62, 83, 84]), with 2 discontinuations for polyuria and urinary tract infection [85] or diabetic ketoacidosis (in the context of temporary lack of access to adequate insulin supply). Ketonaemia was reported stable in several reports when tracked on initiation of therapy [44, 62]. Reported effects on urinary calcium excretion were inconsistent, with both increase and decrease described on treatment [62, 86].

Beyond conventional anti-diabetic therapy, several more specialised pharmacotherapies have been used. Most prominent is rhIGF-1, used since the early 1990s [50]. rhIGF-1 was given to 34.5% of patients, sometimes combined with IGFBP3 [55, 87], or administered as continuous infusion [36, 51, 65, 67] in view of evidence of short IGF-1 half-life in biallelic insulin receptoropathy [88]. Dosing varied from 50 to >1,000 mg/kg/day. Evidence supporting rhIGF-1 use has been reviewed elsewhere [17, 77, 79, 89] and is of relatively low quality despite its frequent use. Outcomes were mixed: clear benefit in 7, partial or temporary benefit in 11, non-response in 4, and 6 discontinuations for adverse events. Illustrative responders showed marked improvements in acanthosis nigricans, insulin, and HbA1c [78, 90]. Adverse events reported during rhIGF-1 therapy include DKA and endometrial carcinoma [91], worsening or development of cardiomyopathy (n^3^4), hypoglycaemia [77], infection, accelerated microvascular complications on abrupt fall in HbA1c [43], airway-compromising tonsillar hypertrophy [36], papillary thyroid cancer (follow-up of [55]) and ovarian enlargement, torsion or tumour (e.g. [51]). Importantly, however, all these complications also occurred in multiple, reportedly rhIGF-1-naïve patients, so attribution to therapy is not established from these data, and indeed cardiomyopathy also reportedly improved during continuous infusion of rhIGF-1 in at least 2 individuals [65, 67].

Another specialised therapy reported was metreleptin, reported in 11 individuals. Modest HbA1c improvement but also non-responses were noted [77, 92], and in a phenotype characterised by leanness the weight loss induced may be undesirable; some patients discontinued metreleptin for this reason [77]. Other adjunctive or exploratory therapies reported included growth hormone with or without concomitant rhIGF-1, which appeared to worsen glycaemia, ursodeoxycholic acid for cholestasis, propranolol for cardiac hypertrophy, and antihypertensives. Suppressive doses of levothyroxine for thyroid carcinoma led to a dramatic improvement in diabetes control with discontinuation of high-dose insulin in one patient [93], but a subsequent small clinical trial of liothyronine did not demonstrate benefit [94].

## DISCUSSION

This study assembles the largest group of individuals with biallelic *INSR*-related IR to date, including 226 unrelated probands carrying 210 distinct *INSR* variants, unifying and extending 40 years of reports. We clarify the mutational spectrum, offer the definitive description of the associated multi-system pathology, and use the scale of the aggregated outcome data to support data-driven prognostication. We also address management questions, including how to conceptualise the complex roles of IGF-1 in pathogenesis and therapy.

The mutational spectrum of biallelic *INSR* disease is broad and largely private, with even recurrent alleles — e.g. S350L, P220L, I146M, T937M, R141W — accounting for only a small minority of cases. This means that genotype-based prediction usually rests on the functional class of novel variants rather than prior experience of specific alleles. Biallelic disease was strongly skewed towards severely hypomorphic or null alleles, distributed in proportion to IC and EC domain length, as expected of a recessive LoF disorder. This is distinct from monoallelic *INSR*-related IR, which features enrichment for IC dominant negative missense variants (companion monoallelic manuscript already submitted to *Genome Medicine*).

The complete “KO” genotype – which we define as the presence of biallelic convincing complete LoF alleles - defines the upper phenotypic severity bound of the disorder. KO individuals cluster at the lowest birthweight SDS and die in infancy, with no KO individual surviving beyond 36 months. In contrast, many homozygotes for missense variants with residual activity are born less growth-restricted and survive into second and third decades, with a continuum of severity seen.

The historical division of INSRopathy into Donohue and Rabson Mendenhall syndromes, and Type A IR was imprecise. Our RSF model shows that continuous risk-based prognostication is feasible, with good discrimination and reasonable calibration. Stratification into risk tertiles suggests potential correlates for these eponymous labels. Before this is formalised, however, prospective data are needed. The model is probabilistic and provisional, trained on retrospective data with wide confidence intervals, and will require external validation and refinement as prospective data accrue. We propose making the RSF model accessible to clinicians for prognostic counselling and to allow adjustment for expected baseline severity when interpreting treatment effects.

The variables driving the RSF model reinforce that the strongest single predictor is KO status. Birthweight SDS ranked next, in keeping with foetal growth restriction being an integrated readout of receptor function, followed by early-onset coagulopathy (likely a marker of severe hepatic involvement), summed CADD score as a continuous index of allelic severity, sex, and liver pathology. The unified experimental function score did not contribute to the variables driving the model, presumably outcompeted by the summed CADD score. This likely reflects that fact that the experimental score was only partially experimental, imputing gaps in the extracellular domain, and making simplistic assumptions about intracellular missense mutation function scores. Rerunning the RSF process when a full set of truly experimental function scores will be of great interest.

Sex appears as a driver due to excess male mortality. This was a surprise given the severe, hormone-responsive ovarian pathology (enlargement, torsion, JGCT, oestrogen-responsive malignancy) seen in numerous affected females. However beyond insulin receptoropathy, male neonates have a general mortality disadvantage of approximately 1.3:1 across virtually all neonatal conditions, including prematurity, sepsis, and undernutrition [95, 96]. They also have higher metabolic rates, faster growth velocity and smaller fat reserves than females [97]. This baseline metabolic and energetic difference may render male infants more susceptible to physiological decompensation in the face of defective *INSR* function. Another non-mutually-exclusive possibility is that ovary-derived oestrogen has a cytoprotective effect. Oestrogen preserves mitochondrial integrity during metabolic stress, localising to mitochondria and improving skeletal muscle bioenergetics independent of ERα signalling [98]. It also extends survival in mice with cardiac-specific deletion of IRS1 and IRS2, and protects pancreatic β-cells from oxidative-stress-induced apoptosis via Erα. These speculative explanations require formal testing in future, however.

This series confirms the extreme IR of biallelic INSRopathy, with early life fasting hypoglycaemia and postprandial hyperglycaemia evolving to sustained hyperglycaemia and early microvascular complications. The confirmed rarity of ketoacidosis, even in KO individuals and in the face of profound hyperglycaemia, suggests that residual insulin-like action mediated by extreme hyperinsulinaemia is sufficient to restrain ketogenesis even when it cannot normalise glucose. Ketoacidosis, when it does occur, tends to accompany intercurrent illness. Despite extreme IR, overt dyslipidaemia was rare and mild or transitory, and MASLD was documented in only 2 patients. This is in sharp contrast to recessive lipodystrophic severe IR, in which IR is accompanied by hypertriglyceridaemia and near-universal MASLD, but consistent with findings in monoallelic defects in either the insulin receptor [99] or downstream signalling gene *PIK3R1* [100].

Neurodevelopmental impairment, reported in around 30% of the cohort, was strongly associated with a history of neonatal hypoglycaemia. The pattern of deficit and its timecourse was moreover consistent with observations of children with other causes of neonatal hypoglycaemia [101-103], which appears to be the most parsimonious explanation. A contribution from disrupted brain insulin signalling cannot be excluded however, and indeed murine neuronal *Insr* KO has behavioural and metabolic consequences [104].

Susceptibility to infection is a common feature of the disorder, and sepsis was the single commonest contributor to death. Susceptibility is likely multifactorial, with chronic hyperglycaemia, leptin deficiency [105, 106], undernutrition and possible defective immune cell insulin/IGF signalling [107, 108], all likely interacting with stressors such as repeated instrumentation. The practical message is that infection is a leading and partly preventable cause of death, and that glycaemic control, nutritional support and scrupulous infection control are important.

Growth failure, both antenatal and postnatal, was the presenting feature in almost half the cohort, and was more extreme in KO individuals. Intrauterine growth restriction is consistent with insulin, acting through INSR, being a major foetal growth factor. Importantly, patients had co-existence of both linear growth impairment and pseudoacromegaloid overgrowth. Where growth is INSR-dependent (especially of bones, muscle and adipose tissue), loss of INSR causes growth impairment; where growth can be driven through IGF1R by paracrine/autocrine IGF-1 or extreme hyperinsulinaemia, in contrast, high GH may produce local overgrowth. The admixed undergrowth/overgrowth phenotype may thus be a reflection of differential tissue-specific insulin/GH/IGF1R crosstalk.

The ovary is a florid example of this overgrowth, giving rise to major morbidity and mortality, including two ovarian torsions, two histologically confirmed JGCT (one bilateral in a neonate), at least seven oophorectomies, and secondary oestrogen-responsive malignancies (two endometrial and one breast carcinoma before 30 years). Marked ovarian enlargement occurs even in KO patients, arguing that much of the ovarian response is IGF1R-mediated. Ovarian stimulation requires intact gonadotrophins, as GnRH suppression abolishes extreme hyperandrogenaemia [109, 110]. Whether early GnRH agonist treatment in severely affected female infants will mitigate ovarian enlargement is worthy of testing, although potential cytoprotective effects of ovarian oestrogen also need be evaluated.

The range of other organ-specific pathology seen in biallelic insulin receptoropathy is well described in this study. This includes nephrocalcinosis and sometimes renal tubulopathy, cardiac hypertrophy and/or left to right shunts, and liver disease, which was present in around half of all patients with data, being far more common in KO individuals. The pattern of *INSR*-related liver pathology — intrahepatic canalicular involvement with inspissated bile and a grossly normal extrahepatic tree — was in keeping with a pattern documented in the index report in 1954 [14], which described inspissated canalicular bile plugs. Bile duct proliferation, and, in longer-surviving cases, portal and periportal fibrosis are added to the recognised trajectory by the current report. This liver pathology phenocopies inherited bile-secretory cholestasis, best exemplified by progressive familial intrahepatic cholestasis [111]. This circumstantially implicates a defect of bile-secretory or ductal machinery in pathogenesis of this characteristic feature of severe biallelic INSRopathy.

The cohort's two cases of diffuse colonic polyposis, together with the marked colonic hypertrophy and prolapse seen almost exclusively in KO individuals, invite a parallel with the epidemiological association between hyperinsulinaemia and colorectal neoplasia [112, 113]. The rare disease association suggests that hyperinsulinaemia is a trophic stimulus to the colonic epithelium, and that this may not be mediated by the INSR. A similar effect may be at play, milder but sustained through adulthood, in common IR, potentially accelerating cancer development and/or growth.

Whether renal and cardiac features of human *INSR* KO or near KO reflect tissue-autonomous developmental and trophic roles of the INSR, the associated severely hyperinsulinaemic endocrine milieu, or grossly perturbed metabolic dialogue among metabolic organs, or a combination of these, is unknown. The relevant tissue-specific *Insr* KOs in mice, which replicate the tissue autonomous signalling defect but not the systemic perturbation, do not replicate the human pathology [114-117].

Recommendations for clinical care of patients with recessive insulin receptoropathy are limited by low-quality evidence from case reports and series. Moreover, insufficient knowledge of natural history makes distinguishing treatment benefits or effects from spontaneous changes difficult. Given these limitations, all conclusions presented here regarding therapies should be taken as observations and not the product of controlled comparisons.

Insulin, sometimes at extreme doses, and metformin remain the backbone of glycaemic management, aimed at reducing hyperglycaemia and its microvascular and infective consequences rather than at correcting the underlying resistance, which no available drug does. Of the newer options, SGLT2i are mechanistically attractive because they lower glucose by a route that is independent of the insulin receptor and so should retain efficacy however severe the receptor defect. The cohort experience, though small, is encouraging: benefit was commented on in 10 of 12 treated individuals. These drugs promote ketogenesis, however, and could precipitate ketoacidosis in a population with limited insulin reserve (indeed one discontinuation followed DKA). Moreover, the renal and cardiovascular benefits established for this class in type 2 diabetes cannot be assumed to transfer to a disorder with distinct renal and cardiac pathology. Nevertheless, on balance SGLT2i are the most promising insulin-independent pharmacological option at present and warrant systematic, prospective evaluation with careful ketone monitoring, rather than uncritical adoption.

Recombinant human IGF-1 is the treatment that most sharply exposes current incomplete understanding of recessive receptoropathy. It was used in a third of the cohort at widely varying doses, and outcomes were mixed. Thus, while rhIGF-1 is worth trialling, it is not reliably effective, and its benefits, where they occur, are often incomplete and offset by possible harm. Reports of tumour growth (including a JGCT) and of worsening cardiac hypertrophy (four individuals on rhIGF-1) are consistent with agonism at IGF1R, the receptor that plausibly mediates several of the disease's proliferative complications, although these could also reflect the natural history of insulin receptoropathy. A recent series supports the notion that rhIGF-1 benefit is limited at the most severe end of the spectrum [77], though on the other hand the two “KO” patients who survived longest both received infusion of rhIGF-1.

The overarching therapeutic message of the current work is that the field has reached the limit of what aggregated case reports can tell it. Progress now requires prospective, multicentre data collection with predefined outcomes, ideally through an international registry and consensus core outcome set, so that the effect of interventions can be judged against the disorder's variable natural history. The prognostic framework developed is one step towards that end, permitting adjustment for expected severity when comparing treated and untreated patients.

Important limitations apply to this study. It is a retrospective aggregation of published and unpublished cases spanning many decades and centres, with attendant ascertainment and publication bias towards the most severe and instructive cases. Phenotyping is inconsistent across sources and eras, assays differ, and ethnicity is unevenly recorded. Outcome analysis is affected by censoring and by improvements in supportive care, which the reporting-era term in the prognostic model only partly captures. Functional consequences were largely inferred from variant class and *in silico* prediction, though the KO definition is stringent.

## CONCLUSIONS

This series defines, at unprecedented scale, the clinical and genetic spectrum of biallelic insulin-receptor disease. The mutational burden is spread evenly across the receptor and in homozygotes forms a graded allelic series with complete KO as the upper severity bound. It is a multi-system disease: beyond profound IR, hypoglycaemia and hyperglycaemia without dyslipidaemia and steatosis, it features severe pre- and postnatal growth failure admixed with widespread soft tissue overgrowth, as well as cholestatic liver disease, renal tubulopathy and nephrocalcinosis, cardiac hypertrophy, ovarian enlargement and colonic overgrowth, with early ovarian or oestrogen-responsive cancers reported. No current agent corrects the receptor defect; insulin and metformin remain the pragmatic mainstay of therapy, with SGLT2 inhibitors an emerging promising insulin-independent option. rhIGF-1 is worth trialling but is of uncertain risk and benefit balance. We present a new prognostic model showing that severity can be quantified from routinely available features, offering a path away from eponymous categories towards individualised, mechanism-anchored risk assessment. The priorities now are prospective, registry-based data with predefined outcomes, external validation of the prognostic framework, so that counselling and evaluation of therapies can rest on firmer ground, and investigation of the basis of increased male vulnerability, and preclinical investigation of novel advanced therapies.

## Supplementary Material

This is a list of supplementary files associated with this preprint. Click to download.

- DSilvaAdditionalFile12.10.26.xlsx
- DSilvaetalAdditionalFile22.10.26.docx
- DSilvaetalAdditionalFile32.10.26.docx

## Figures and Tables

**Figure 1:**
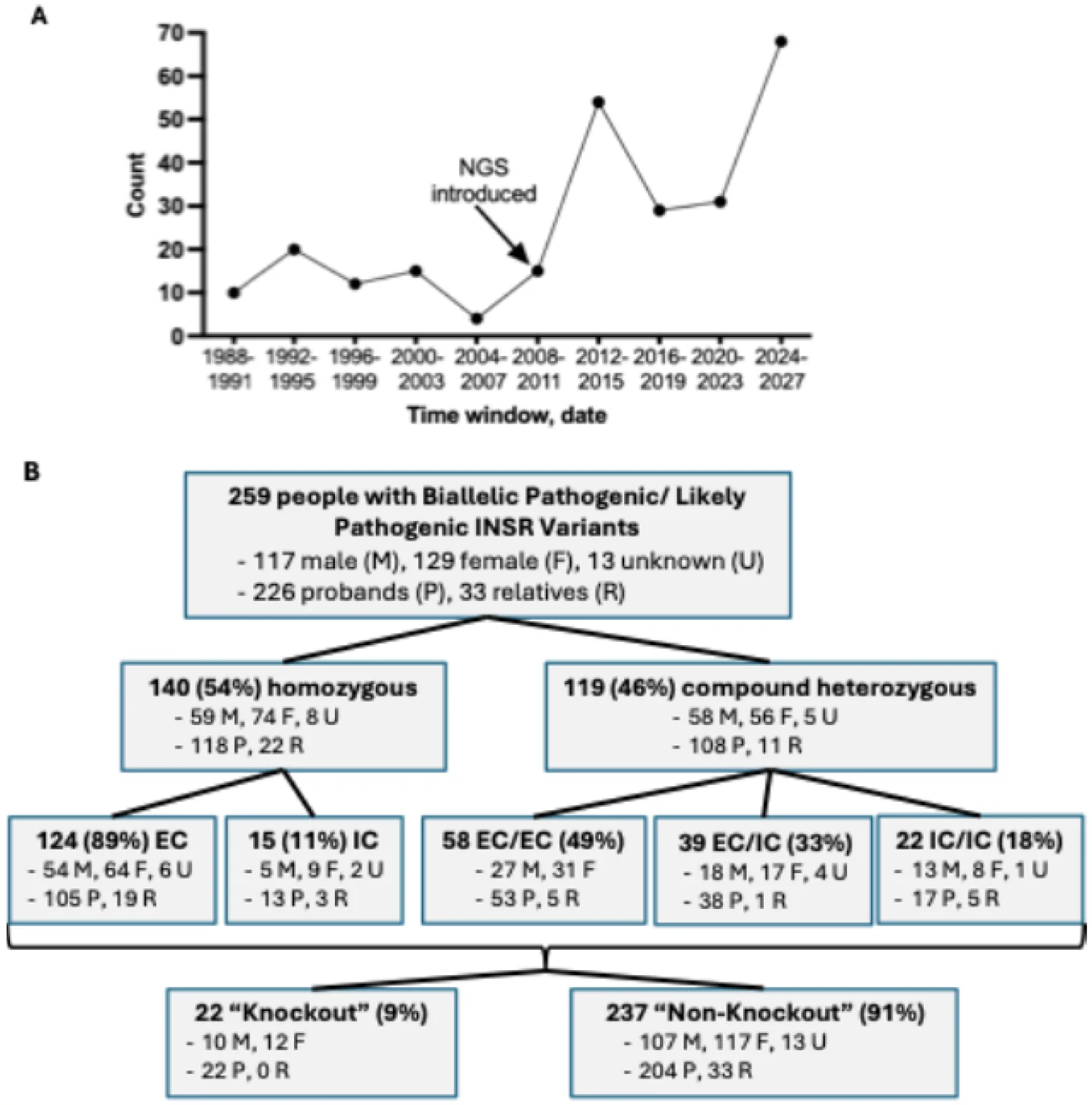
Ascertainment and Composition of INSR Cohort Studied. **A**. Rate of ascertainment of people with biallelic INSR-related insulin resistance, including published and unpublished cases. NGS = next generation sequencing **B**. Composition of cohort reported. EC = extracellular (variant); IC = Intracellular (variant); “Knockout” is used to describe people with two convincing complete loss-of-function variants in *trans*, as described in Methods

**Figure 2:**
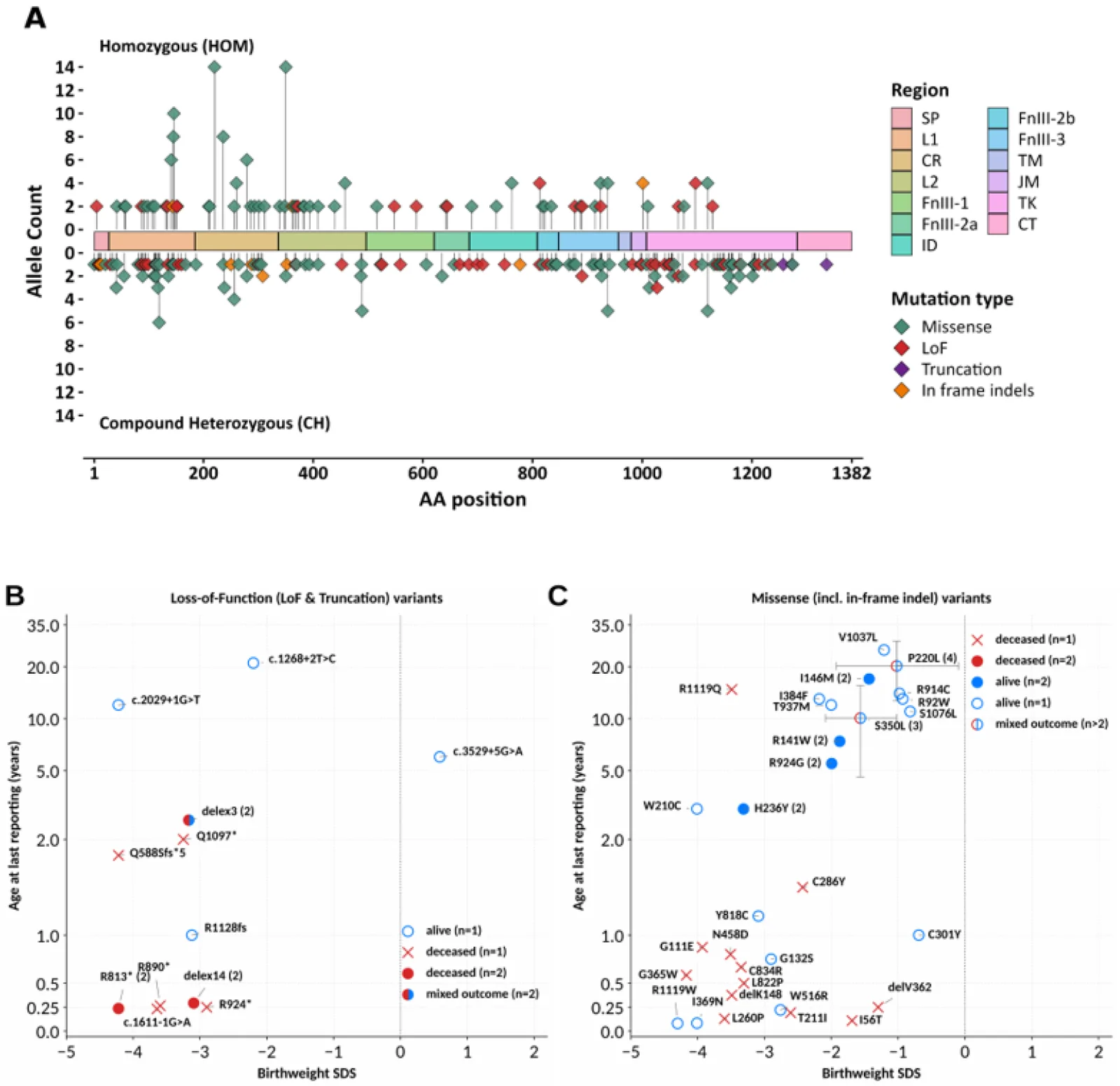
Biallelic INSR Mutational Spectrum and Homozygous Phenotypic Range. **A**. Lollipop plot of variants demonstrated in homozygous (above ribbon) and compound heterozygous (below ribbon) individuals. LoF = loss-of-function (nonsense or frameshift variants predicted to undergo nonsense-mediated decay, or essential splice variants). AA = amino acid. INSR domains are indicated. SP = signal peptide; L1/2 = first/second leucine-rich domains; CR = cysteine rich domain; FnIII = Fibronectin III; ID = insert domain; TM = transmembrane domain; JM = juxtamembrane domain; TK = tyrosine kinase domain; CT = C terminal domain **B,C**. Birthweight standard deviation score (SDS) plotted against age at death or censoring for individuals with homozygous LoF variants (**B**) or homozygous missense variants/in-frame indels (**C**)

**Figure 3:**
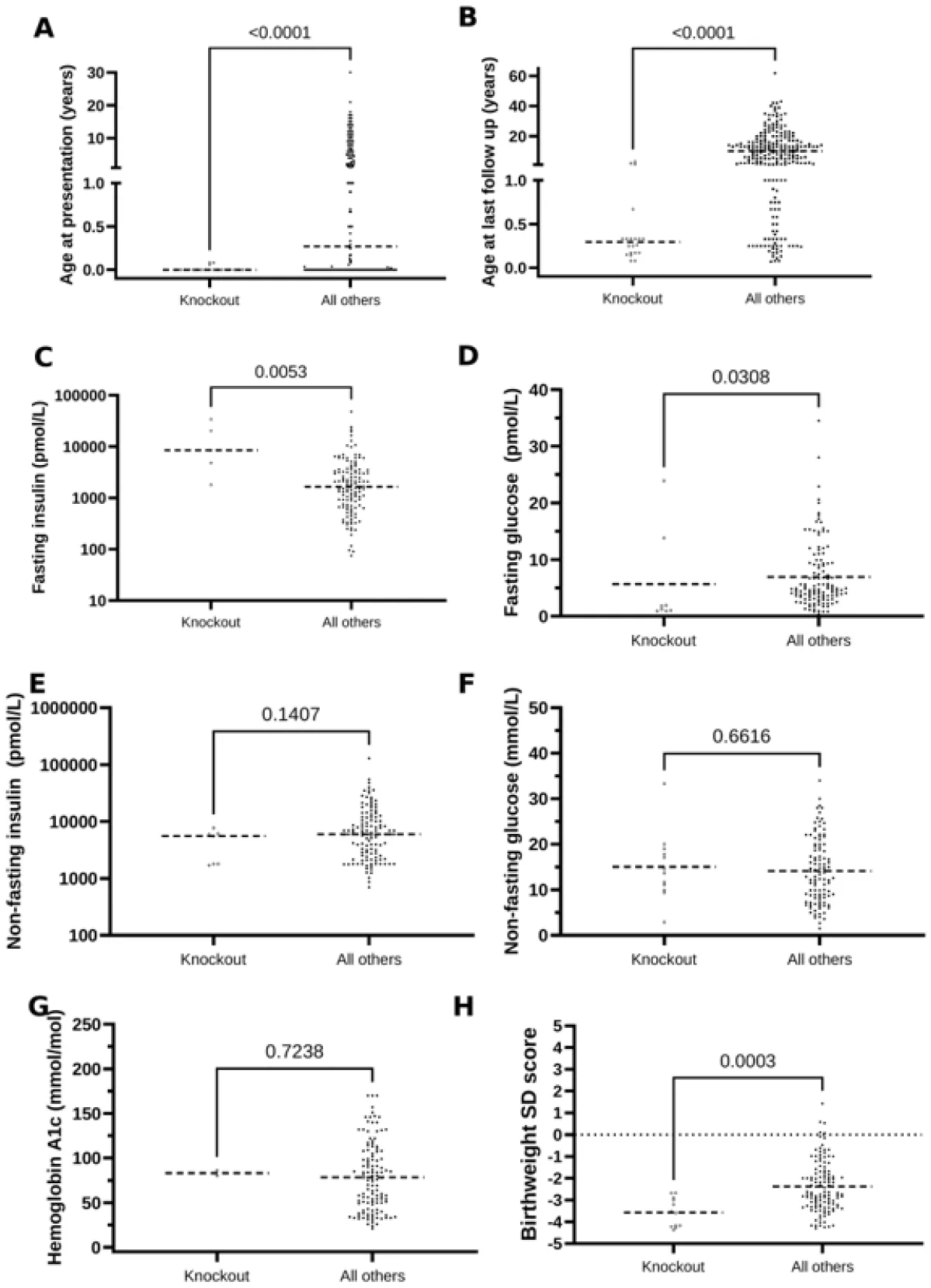
Phenotypic Comparison of Knockout and non-Knockout Individuals. Scatterplots of **A**. Age at presentation, **B**. Age at last follow up, **C**. Fasting plasma insulin concentration, **D**. Fasting blood glucose concentration, **E**. Non-fasting plasma insulin concentration (postprandial or after a specified challenge such as an oral glucose tolerance test), **F**. Non-fasting blood glucose concentration (postprandial or after a specified challenge such as an oral glucose tolerance test), **G**. Haemoglobin A1c and **H**. Birthweight standard deviation (SD) score. Medians are indicated with dashed lines. P values shown relate to the null hypothesis of no difference between the groups indicated (Mann-Whitney test for all but SDS score comparison, which used the one sample Wilcoxon signed rank test)

**Figure 4:**
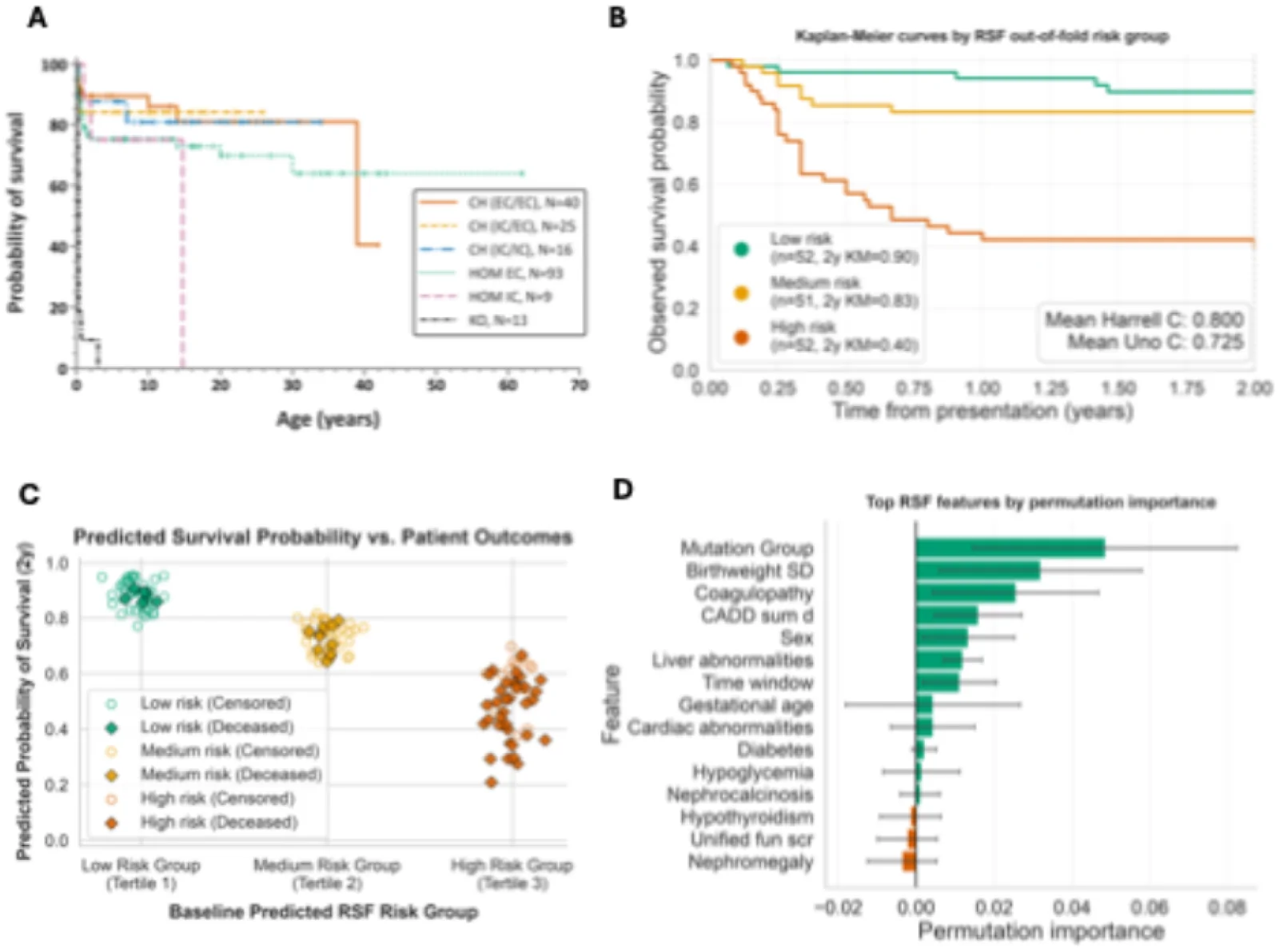
Longevity and Prognostic Modelling in Biallelic Insulin Receptoropathy. **A**. Kaplan-Meier observed survival curves stratified by genotype groups. KO = knockout, as defined in Methods. HOM = homozygous; CH = compound heterozygous. IC and EC denote variant locations in protein, where IC = intracellular; EC = extracellular. **B**. Kaplan-Meier observed survival curves stratified by baseline predicted Risk Score tertiles (Low, Medium, and High Risk groups) derived from Random Survival Forest Modelling, showing distinct trajectory segregation over a 2-year clinical follow up window. **C**. Predicted probability of survival for all individuals in low, medium and high risk tertiles defined by the Random Survival Forest (RSF) model **D**. Permutation feature importance in the RSF Model. Predictors are ordered from highest to lowest mean out-of-fold permutation importance over 30 repeated iterations. Green bars indicate positive predictive contribution to survival discrimination, while orange bars represent negligible or negative predictive value in the context of multi-system interactions. Error bars represent standard deviation across validation folds.

**Figure 5:**
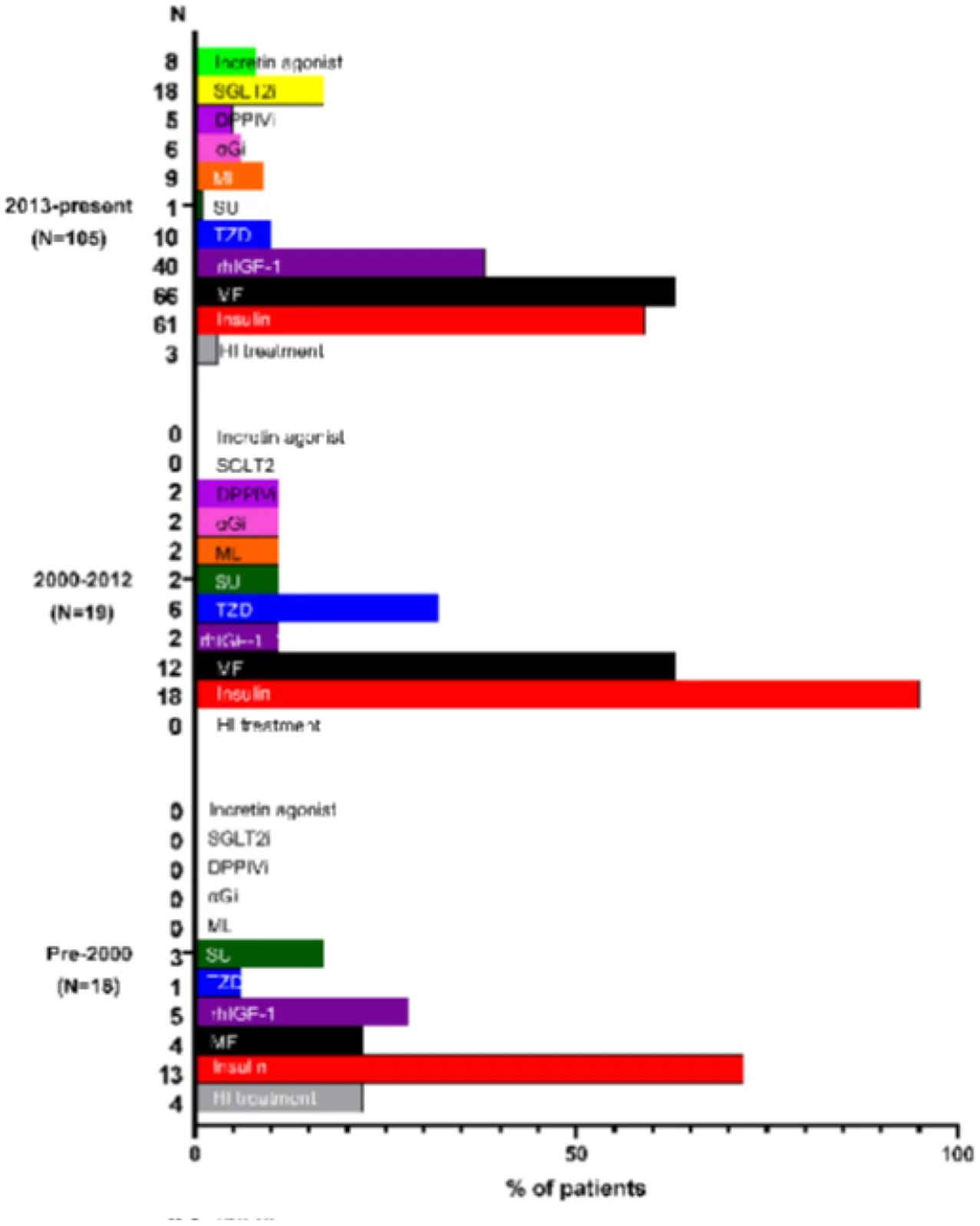
Historical Patterns of Therapy in Biallelic Insulin Receptoropathy. Proportions of individuals treated with different pharmacotherapies across the eras indicated. SGLT2i = sodium-glucose co-transporter 2 inhibitor; DPPIVi = dipeptidyl peptidase IV inhibitor; aGi = a-glucosidase inhibitor; ML = metreleptin; SU = sulphonylurea; TZD = thiazolidinedione; rhIGF-1 = recombinant human insulin-like growth factor 1; MF = metformin; HI = hyperinsulinism

**Table 1:** Frequencies of presenting features. N, number; KO, knock out (indicating complete loss of function variants); ^1^ “Hyperandrogenism” includes polycystic ovary syndrome, hirsutism, or other signs or symptoms of hyperandrogenism and is reported among females only; ^2^ “Hyperinsulinaemia” includes acanthosis nigricans or hyperinsulinaemia; ^3^ “Other” includes cardiomyopathy, developmental delay, nephrocalcinosis, bowel obstruction, and tachypnoea. Many patients had multiple presenting features, thus percentages do not total 100. N for each group indicates the number of patients in whom that reason for presentation was reported. KO vs non KO statistical comparison used a 2-sided Fisher’s exact test.

		Growth Failure	Dysmorphic features	Hyperglycaemia	Hypoglycaemia	Hyperandrogenism^1^	Hyperinsulinaemia^2^	Family screening	Ot
All (N=259)	Yes	97	76	67	27	17	50	7	27
	Total	219	219	219	219	130	219	220	21
	%	44.3	34.7	30.6	12.3	13.1	22.8	3.2	12
KO (N=21)	Yes	17	14	3	4	0	1	0	1
	Total	21	21	18	17	0	20	21	20
	%	81.0	66.7	16.7	23.5	N/A	5.0	0.0	5
non-KO (N=230)	Yes	80	62	64	23	20	49	7	26
	Total	198	198	198	175	198	198	199	19
	%	40.4	31.3	32.3	13.1	10.1	24.7	3.5	13
p (KO vs non-KO)		0.0004	0.0028	0.196	0.268	0.23	0.05	>0.99	0.

**Table 2:** Categorical Clinical Features of Biallelic Insulin Receptoropathy; DKA = diabetic ketoacidosis; Ovary = ovarian involvement; Androgen = clinical or biochemical hyperandrogenism MASLD = metabolic dysfunction-associated steatotic liver disease; Lipids = dyslipidaemia; N = No; Y = Yes; Total = total number of subjects in whom a feature might be present; KO = Knockout, as defined in Methods; Non-KO = other genotypes not meeting KO criteria; Min% = Minimum prevalence estimate (assumes all blanks are No); % = Point prevalence estimate (ignores blanks); Max%, Maximum prevalence (assumes all blanks are Yes); n/a (not applicable: point prevalence estimate could not be calculated due to lack of data)

	All (N=259, 129 female)						non-KO (N=237, 117 female)						KO (N=22, 12 female)				
	Y	N	Total	Min%	%	Max%	Y	N	Total	Min%	%	Max%	Y	N	Total	Min%	%
Glycemic features																	
Hyperglycaemia	157	13	259	60.6	92.4	95.0	140	13	237	59.1	91.5	94.5	17	0	22	77.3	100.0
Hypoglycaemia	103	40	259	39.8	72.0	84.6	87	38	237	36.7	69.6	84.0	16	2	22	72.7	88.9
Retinopathy	20	55	259	7.7	26.7	78.8	20	54	237	8.4	27.0	77.2	0	1	22	0.0	0.0
Nephropathy	30	56	259	11.6	34.9	78.4	29	55	237	12.2	34.5	76.8	1	1	22	4.5	50.0
Neuropathy	10	35	259	3.9	22.2	86.5	10	35	237	4.2	22.2	85.2	0	0	22	0.0	n/a
Islet hyperplasia	8	3	259	3.1	72.7	98.8	7	3	237	3.0	70.0	98.7	1	0	22	4.5	100.0
DKA	16	107	259	6.2	13.0	58.7	15	98	237	6.3	13.3	58.6	1	9	22	4.5	10.0
Overgrowth features																	
Acanthosis nigricans	168	25	259	64.9	87.0	90.3	160	23	237	67.5	87.4	90.3	8	2	22	36.4	80.0
Colon hyperplasia	12	31	259	4.6	27.9	88.0	10	31	237	4.2	24.4	86.9	2	0	22	9.1	100.0
Facial dysmorphism	162	32	259	62.5	83.5	87.6	143	31	237	60.3	82.2	86.9	19	1	22	86.4	95.0
Hypertrichosis	147	13	259	56.8	91.9	95.0	130	13	237	54.9	90.9	94.5	17	0	22	77.3	100.0
Gingival hyperplasia	32	12	259	12.4	72.7	95.4	30	12	237	12.7	71.4	94.9	2	0	22	9.1	100.0
Tonsil hypertrophy	21	7	259	8.1	75.0	97.3	20	7	237	8.4	74.1	97.0	1	0	22	4.5	100.0
Pineal hyperplasia	1	10	259	0.4	9.1	96.1	1	9	237	0.4	10.0	96.2	0	1	22	0.0	0.0
Cancer	6	210	259	2.3	2.8	18.9	4	192	237	1.7	2.0	19.0	2	18	22	9.1	10.0
Abdominal protuberance	79	24	259	30.5	76.7	90.7	65	24	237	27.4	73.0	89.9	14	0	22	63.6	100.0
Heart disease	58	24	259	22.4	70.7	90.7	50	23	237	21.1	68.5	90.3	8	1	22	36.4	88.9
Nephromegaly	44	29	259	17.0	60.3	88.8	40	27	237	16.9	59.7	88.6	4	2	22	18.2	66.7
Dental abnormality	48	7	259	18.5	87.3	97.3	48	7	237	20.3	87.3	97.0	0	0	22	0.0	n/a
Undergrowth features																	
Impaired growth	115	36	259	44.4	76.2	86.1	104	36	237	43.9	74.3	84.8	11	0	22	50.0	100.0
Low adipose / muscle	99	40	259	38.2	71.2	84.6	81	39	237	34.2	67.5	83.5	18	1	22	81.8	94.7
Reproductive features																	
Genital hyperplasia	83	31	259	32.0	72.8	88.0	69	31	237	29.1	69.0	86.9	14	0	22	63.6	100.0
Ovarian enlargement (females only)	55	6	129	42.6	90.2	95.3	51	6	117	43.6	89.5	94.9	4	0	12	33.3	100.0
Hyperandrogenism (females only)	46	13	129	35.7	78.0	89.9	44	12	117	37.6	78.6	89.7	2	1	12	16.7	66.7
Lipid features																	
MASLD	2	36	259	0.8	5.3	86.1	2	34	237	0.8	5.6	85.7	0	2	22	0.0	0.0
Dyslipidaemia	6	33	259	2.3	15.4	87.3	6	30	237	2.5	16.7	87.3	0	3	22	0.0	0.0
Other features																	
Neurocognitive	25	55	259	9.7	31.3	78.8	24	52	237	10.1	31.6	78.1	1	3	22	4.5	25.0
Frequent infections	31	12	259	12.0	72.1	95.4	27	12	237	11.4	69.2	94.9	4	0	22	18.2	100.0
Liver disease	56	46	259	21.6	54.9	82.2	48	45	237	20.3	51.6	81.0	8	1	22	36.4	88.9
Coagulopathy	9	4	259	3.5	69.2	98.5	8	4	237	3.4	66.7	98.3	1	0	22	4.5	100.0
Nephrocalcinosis	61	20	259	23.6	75.3	92.3	59	18	237	24.9	76.6	92.4	2	2	22	9.1	50.0

## Abbreviations

AUC: Area under the (receiver operating characteristic) curve
BMI: Body mass index
CADD: Combined Annotation-Dependent Depletion
CDC: Centers for Disease Control and Prevention
CI: Confidence interval
DKA: Diabetic ketoacidosis
DPPIV: Dipeptidyl peptidase IV
DR: Density ratio
EC: Extracellular (domain)
ERα: Oestrogen receptor alpha
GDM: Gestational diabetes mellitus
GH: Growth hormone
GLP-1: Glucagon-like peptide-1
gnomAD: Genome Aggregation Database
GnRH: Gonadotropin-releasing hormone
HbA1c: Glycated haemoglobin
HDL-C: High-density lipoprotein cholesterol
IC: Intracellular (domain)
IGF-1: Insulin-like growth factor 1
IGF1R: Insulin-like growth factor 1 receptor
IGFBP3: Insulin-like growth factor binding protein 3
*INSR*: Insulin receptor (gene if italic; protein if non italic)
IR: Insulin resistance
IRS1/IRS2: Insulin receptor substrate 1 / 2
IUGR: Intrauterine growth restriction
JGCT: Juvenile granulosa cell tumour
KO: Knockout
LDL-C: Low-density lipoprotein cholesterol
LoF: Loss of function
MANE: NCBI Matched Annotation from NCBI and EBI (transcript set, Methods)
MASLD: Metabolic dysfunction-associated steatotic liver disease
MAVE: Massively multiplexed assay of variant effect
NGS: Next Generation Sequencing
NMD: Nonsense-mediated (mRNA) decay
OR: Odds ratio
PDR: Proliferative diabetic retinopathy
PIK3R1: Phosphoinositide-3-kinase regulatory subunit 1 (gene symbol, one mention)
PTC: Papillary thyroid cancer
rhIGF-1: Recombinant human insulin-like growth factor 1
RSF: Random Survival Forest
SD(S): Standard Deviation (Score)
SGLT2: Sodium-glucose cotransporter 2

## References

[1] White MF, Kahn CR. Insulin action at a molecular level – 100 years of progress. Mol Metab. 2021;52:101304. 10.1016/j.molmet.2021.101304 PMC8551477 34274528

[2] Kadowaki T, Bevins CL, Cama A, Ojamaa K, Marcus-Samuels B, Kadowaki H, Beitz L, McKeon C, Taylor SI. Two mutant alleles of the insulin receptor gene in a patient with extreme insulin resistance. Sci (New York NY). 1988;240:787–90. 10.1126/science.2834824 2834824

[3] Yoshimasa Y, Seino S, Whittaker J, Kakehi T, Kosaki A, Kuzuya H, Imura H, Bell GI, Steiner DF. Insulin-resistant diabetes due to a point mutation that prevents insulin proreceptor processing. Sci (New York NY). 1988;240:784–7. 10.1126/science.3283938 3283938

[4] Ebina Y, Ellis L, Jarnagin K, Edery M, Graf L, Clauser E, Ou JH, Masiarz F, Kan YW, Goldfine ID. The human insulin receptor cDNA: the structural basis for hormone-activated transmembrane signalling. Cell. 1985;40:747–58. 10.1016/0092-8674(85)90334-4 2859121

[5] Ullrich A, Bell JR, Chen EY, Herrera R, Petruzzelli LM, Dull TJ, Gray A, Coussens L, Liao YC, Tsubokawa M. Human insulin receptor and its relationship to the tyrosine kinase family of oncogenes. Nature. 1985;313:756–61. 10.1038/313756a0 2983222

[6] Taylor SI, Cama A, Accili D, Barbetti F, Quon MJ, de la Luz Sierra M, Suzuki Y, Koller E, Levy-Toledano R, Wertheimer E. Mutations in the insulin receptor gene. Endocr Rev. 1992;13:566–95. 10.1210/edrv-13-3-566 1330507

[7] Taylor SI. Lilly Lecture: molecular mechanisms of insulin resistance. Lessons from patients with mutations in the insulin-receptor gene. Diabetes. 1992;41:1473–90. 10.2337/diab.41.11.1473 1327927

[8] Mesika A, Klar A, Falik Zaccai TC. INSR-Related Severe Insulin Resistance Syndrome. GeneReviews® [Internet]. Seattle: University of Washington; 2018. 29369573

[9] Whittaker J, Soos MA, Siddle K. Hybrid insulin receptors. Molecular mechanisms of negative-dominant mutations in receptor-mediated insulin resistance. Diabetes Care. 1990;13:576–81. 10.2337/diacare.13.6.576 2192845

[10] Cama A, Quon MJ, de la Luz Sierra M, Taylor SI. Substitution of isoleucine for methionine at position 1153 in the beta-subunit of the human insulin receptor. A mutation that impairs receptor tyrosine kinase activity, receptor endocytosis, and insulin action. J Biol Chem. 1992;267:8383–9. 1314826

[11] Treadway JL, Morrison BD, Soos MA, Siddle K, Olefsky J, Ullrich A, McClain DA, Pessin JE. Transdominant inhibition of tyrosine kinase activity in mutant insulin/insulin-like growth factor I hybrid receptors. Proc Natl Acad Sci USA. 1991;88:214–8. 10.1073/pnas.88.1.214 PMC50780 1846039

[12] Frattali AL, Treadway JL, Pessin JE. Transmembrane signaling by the human insulin receptor kinase. Relationship between intramolecular beta subunit trans- and cis-autophosphorylation and substrate kinase activation. J Biol Chem. 1992;267:19521–8. 1326556

[13] Grako KA, McClain DA, Olefsky JM. Hybrid formation between endogenous mouse and transfected human tyrosine kinase-deficient (A/K1018) insulin receptors leads to decreased insulin sensitivity in 3T3-L1 adipocytes. Mol Endocrinol (Baltimore Md). 1994;8:682–92. 10.1210/mend.8.6.7935484 7935484

[14] Donohue WL, Uchida I. Leprechaunism: a euphuism for a rare familial disorder. J Pediatr. 1954;45:505–19. 10.1016/s0022-3476(54)80113-2 13212592

[15] Rabson SM, Mendenhall EN. Familial hypertrophy of pineal body, hyperplasia of adrenal cortex and diabetes mellitus; report of 3 cases. Am J Clin Pathol. 1956;26:283–90. 10.1093/ajcp/26.3.283 13302174

[16] Kahn CR, Flier JS, Bar RS, Archer JA, Gorden P, Martin MM, Roth J. The syndromes of insulin resistance and acanthosis nigricans. Insulin-receptor disorders in man. N Engl J Med. 1976;294:739–45. 10.1056/NEJM197604012941401 176581

[17] Semple RK, Patel KA, Auh S, ADA/EASD Precision Medicine Working Group, Brown RJ. Genotype-stratified treatment for monogenic insulin resistance: a systematic review. Commun Med. 2023;3:134. 10.1038/s43856-023-00368-9 PMC10550936 37794082

[18] Karczewski KJ, Francioli LC, Tiao G, Cummings BB, Alföldi J, Wang Q, Collins RL, Laricchia KM, Ganna A, Birnbaum DP, The mutational constraint spectrum quantified from variation in 141,456 humans. Nature. 2020;581:434–43. 10.1038/s41586-020-2308-7 PMC7334197 32461654

[19] Lindeboom RGH, Vermeulen M, Lehner B, Supek F. The impact of nonsense-mediated mRNA decay on genetic disease, gene editing and cancer immunotherapy. Nat Genet. 2019;51:1645–51. 10.1038/s41588-019-0517-5 PMC6858879 31659324

[20] Jaganathan K, Kyriazopoulou Panagiotopoulou S, McRae JF, Darbandi SF, Knowles D, Li YI, Kosmicki JA, Arbelaez J, Cui W, Schwartz GB, Predicting Splicing from Primary Sequence with Deep Learning. Cell. 2019;176:535–e548524. 10.1016/j.cell.2018.12.015 30661751

[21] Schubach M, Maass T, Nazaretyan L, Roner S, Kircher M. CADD v1.7: using protein language models, regulatory CNNs and other nucleotide-level scores to improve genome-wide variant predictions. Nucleic Acids Res. 2024;52:D1143–54. 10.1093/nar/gkad989 PMC10767851 38183205

[22] Kircher M, Witten DM, Jain P, O'Roak BJ, Cooper GM, Shendure J. A general framework for estimating the relative pathogenicity of human genetic variants. Nat Genet. 2014;46:310–5. 10.1038/ng.2892 PMC3992975 24487276

[23] Cannon S, Williams M, Gunning AC, Wright CF. Evaluation of in silico pathogenicity prediction tools for the classification of small in-frame indels. BMC Med Genomics. 2023;16:36. 10.1186/s12920-023-01454-6 PMC9972633 36855133

[24] Aslanzadeh V, Brierley GV, Kumar R, Çubuk H, Vigouroux C, Matreyek KA, Kudla G, Semple RK. Deep mutational scanning of the human insulin receptor ectodomain to inform precision therapy for insulin resistance. Nat Commun. 2025;16:9143. 10.1038/s41467-025-64178-4 PMC12528385 41093840

[25] Villar J, Cheikh Ismail L, Victora CG, Ohuma EO, Bertino E, Altman DG, Lambert A, Papageorghiou AT, Carvalho M, Jaffer YA, International standards for newborn weight, length, and head circumference by gestational age and sex: the Newborn Cross-Sectional Study of the INTERGROWTH-21st Project. Lancet (London England). 2014;384:857–68. 10.1016/S0140-6736(14)60932-6 25209487

[26] Centers for Disease Control and Prevention, National Center for Health Statistics. National Health and Nutrition Examination Survey Data, 2011–2018. Hyattsville, MD: U.S. Department of Health and Human Services. Available at: https://www.cdc.gov/nchs/nhanes/

[27] Collin-Chavagnac D, Saint-Martin C, Bedidi L, Lebreton L, Aslanzadeh V, Vigouroux C, Bellanné-Chantelot C, Semple RK, Lascols O, Jéru I. Insulin receptor variants: Extending the traditional Mendelian spectrum. Genet medicine: official J Am Coll Med Genet. 2025;27:101404. 10.1016/j.gim.2025.101404 40094207

[28] Hassan SS, Musa SA, De Franco E, Myers R, Van Heugten R, Babiker OO, Ibrahim AA, MohamadSalih GF, Ahmed A, Shatta JA, Characterization of monogenic diabetes among Sudanese children: a multi-center experience from a population with high consanguinity. J Pediatr Endocrinol metabolism: JPEM. 2026;39:65–75. 10.1515/jpem-2025-0316 PMC12780942 41275391

[29] Maassen JA, Tobias ES, Kayserilli H, Tukel T, Yuksel-Apak M, D'Haens E, Kleijer WJ, Féry F, van der Zon GC. Identification and functional assessment of novel and known insulin receptor mutations in five patients with syndromes of severe insulin resistance. J Clin Endocrinol Metab. 2003;88:4251–7. 10.1210/jc.2003-030034 12970295

[30] Aftab S, Shaheen T, Asif R, Anjum MN, Saeed A, Manzoor J, Cheema HA. Management challenges of Rabson Mendenhall syndrome in a resource limited country: a case report. J Pediatr Endocrinol metabolism: JPEM. 2022;35:1429–32. 10.1515/jpem-2022-0214 36106528

[31] Roach P, Zick Y, Formisano P, Accili D, Taylor SI, Gorden P. A novel human insulin receptor gene mutation uniquely inhibits insulin binding without impairing posttranslational processing. Diabetes. 1994;43:1096–102. 10.2337/diab.43.9.1096 8070609

[32] Krook A, Kumar S, Laing I, Boulton AJ, Wass JA, O'Rahilly S. Molecular scanning of the insulin receptor gene in syndromes of insulin resistance. Diabetes. 1994;43:357–68. 10.2337/diab.43.3.357 8314008

[33] Raffan E, Soos MA, Rocha N, Tuthill A, Thomsen AR, Hyden CS, Gregory JW, Hindmarsh P, Dattani M, Cochran E, Founder effect in the Horn of Africa for an insulin receptor mutation that may impair receptor recycling. Diabetologia. 2011;54:1057–65. 10.1007/s00125-011-2066-z PMC3071941 21318406

[34] al-Gazali LI, Khalil M, Devadas K. A syndrome of insulin resistance resembling leprechaunism in five sibs of consanguineous parents. J Med Genet. 1993;30:470–5. 10.1136/jmg.30.6.470 PMC1016418 8326490

[35] Hone J, Accili D, al-Gazali LI, Lestringant G, Orban T, Taylor SI. Homozygosity for a new mutation (Ile119–>Met) in the insulin receptor gene in five sibs with familial insulin resistance. J Med Genet. 1994;31:715–6. 10.1136/jmg.31.9.715 PMC1050083 7815442

[36] Abe Y, Sato T, Takagi M, Watanabe T, Nagayama Y, Hasegawa T, Abe T. A case of Rabson-Mendenhall syndrome with a novel mutation in the tyrosine kinase domain of the insulin receptor gene complicated by medullary sponge kidney. J Pediatr Endocrinol metabolism: JPEM. 2012;25:587–90. 10.1515/jpem-2011-0473 22876563

[37] Takeuchi T, Ishigaki Y, Hirota Y, Hasegawa Y, Yorifuji T, Kadowaki H, Akamizu T, Ogawa W, Katagiri H. Clinical characteristics of insulin resistance syndromes: A nationwide survey in Japan. J diabetes Invest. 2020;11:603–16. 10.1111/jdi.13171 PMC7232299 31677333

[38] Kadowaki H, Takahashi Y, Ando A, Momomura K, Kaburagi Y, Quin JD, MacCuish AC, Koda N, Fukushima Y, Taylor SI, Four mutant alleles of the insulin receptor gene associated with genetic syndromes of extreme insulin resistance. Biochem Biophys Res Commun. 1997;237:516–20. 10.1006/bbrc.1997.7181 9299395

[39] Kawashima Y, Nishimura R, Utsunomiya A, Kagawa R, Funata H, Fujimoto M, Hanaki K, Kanzaki S. Leprechaunism (Donohue syndrome): a case bearing novel compound heterozygous mutations in the insulin receptor gene. Endocr J. 2013;60:107–12. 10.1507/endocrj.ej12-0289 22972224

[40] Amaratunga SA, Hussein Tayeb T, Muhamad Sediq RN, Hama Salih FK, Dusatkova P, Wakeling MN, De Franco E, Pruhova S, Lebl J. Paediatric diabetes subtypes in a consanguineous population: a single-centre cohort study from Kurdistan, Iraq. Diabetologia. 2024;67:113–23. 10.1007/s00125-023-06030-2 PMC10709478 37897565

[41] Bastaki F, Nair P, Mohamed M, Khadora MM, Saif F, Tawfiq N, Al-Ali MT, Hamzeh AR. Identification of a Novel Homozygous INSR Variant in a Patient with Rabson-Mendenhall Syndrome from the United Arab Emirates. Hormone Res paediatrics. 2017;87:64–8. 10.1159/000447090 27326825

[42] Al-Kandari H, Al-Abdulrazzaq D, Al-Jaser F, Al-Mulla F, Davidsson L. Rabson-Mendenhall Syndrome in a brother-sister pair in Kuwait: Diagnosis and 5 year follow up. Prim Care Diabetes. 2021;15:175–7. 10.1016/j.pcd.2020.07.012 32843252

[43] Nakashima N, Umeda F, Yanase T, Nawata H. Insulin resistance associated with substitution of histidine for arginine 252 in the alpha-subunit of the human insulin receptor: trial of insulin-like growth factor I injection therapy to enhance insulin sensitivity. J Clin Endocrinol Metab. 1995;80:3662–7. 10.1210/jcem.80.12.8530617 8530617

[44] Dos Santos SS, Ramaldes LA, Gabbay MAL, Moises RCS, Dib SA. Use of a Sodium-Glucose Cotransporter 2 Inhibitor, Empagliflozin, in a Patient with Rabson-Mendenhall Syndrome. Hormone Res paediatrics. 2021;94:313–6. 10.1159/000519613 34551418

[45] Robinson C, Cochran E, Gorden P, Brown RJ. Management of Diabetic Ketoacidosis in Severe Insulin Resistance. Diabetes Care. 2016;39:e116–118. 10.2337/dc16-0635 PMC4955933 27271188

[46] Musso C, Cochran E, Moran SA, Skarulis MC, Oral EA, Taylor S, Gorden P. Clinical course of genetic diseases of the insulin receptor (type A and Rabson-Mendenhall syndromes): a 30-year prospective. Medicine. 2004;83:209–22. 10.1097/01.md.0000133625.73570.54 15232309

[47] Lim K, Haider A, Adams C, Sleigh A, Savage DB. Lipodistrophy: a paradigm for understanding the consequences of overloading adipose tissue. Physiol Rev. 2021;101:907–93. 10.1152/physrev.00032.2020 33356916

[48] Nobile S, Semple RK, Carnielli VP. A novel mutation of the insulin receptor gene in a preterm infant with Donohue syndrome and heart failure. J Pediatr Endocrinol metabolism: JPEM. 2012;25:363–6. 10.1515/jpem-2011-0448 22768670

[49] Dozio N, Scavini M, Beretta A, Sartori S, Meschi F, Sarugeri E, Pozza G. In vivo metabolic effects of insulin-like growth factor-I not mediated through the insulin receptor. J Clin Endocrinol Metab. 1995;80:1325–8. 10.1210/jcem.80.4.7714106 7714106

[50] Nakashima N, Miyamura T, Yamashita T, Yamauchi T, Umeda F, Kawada Y, Noda M, Nawata H. Type A-insulin resistance with lipopexia on extremities: a case report. Endocrinologia japonica. 1992;39:347–53. 10.1507/endocrj1954.39.347 1446650

[51] Weber DR, Stanescu DE, Semple R, Holland C, Magge SN. Continuous subcutaneous IGF-1 therapy via insulin pump in a patient with Donohue syndrome. J Pediatr Endocrinol metabolism: JPEM. 2014;27:1237–41. 10.1515/jpem-2013-0402 PMC4535795 25153212

[52] Longo N, Wang Y, Smith SA, Langley SD, DiMeglio LA, Giannella-Neto D. Genotype-phenotype correlation in inherited severe insulin resistance. Hum Mol Genet. 2002;11:1465–75. 10.1093/hmg/11.12.1465 12023989

[53] Brisigotti M, Fabbretti G, Pesce F, Gatti R, Cohen A, Parenti G, Callea F. Congenital bilateral juvenile granulosa cell tumor of the ovary in leprechaunism: a case report. Pediatr Pathol. 1993;13:549–58. 10.3109/15513819309048242 8247952

[54] Casati S, Zoppini G, Muggeo M, Marchini G. Sustained regression of florid diabetic retinopathy in a patient with Donohue syndrome (leprechaunism). Eur J Ophthalmol. 2010;20:224–7. 10.1177/112067211002000133 19882513

[55] de Kerdanet M, Caron-Debarle M, Nivot S, Gaillot T, Lascols O, Fremont B, Bonaure M, Gie S, Massart C, Capeau J. Ten-year improvement of insulin resistance and growth with recombinant human insulin-like growth factor 1 in a patient with insulin receptor mutations resulting in leprechaunism. Diabetes Metab. 2015;41:331–7. 10.1016/j.diabet.2014.11.001 25465274

[56] Globa E, Christesen HT, Mortensen MB, Houghton JAL, Nielsen AL, Detlefsen S, Flanagan SE. Congenital hyperinsulinism in the Ukraine: a 10-year national study. Front Endocrinol. 2024;15:1497579. 10.3389/fendo.2024.1497579 PMC11686448 39741883

[57] Kushchayeva Y, Vasko V, Jensen K, Mendonca Torres MC, Dwyer JE, Auh S, Startzell M, Kushchayev S, Abel BS, Skarulis M, Differentiated Thyroid Cancer in Severe Insulin Resistance. AACE Endocrinol Diabetes. 2025;12:229–40. 10.1016/j.aed.2025.08.015 PMC12744789 41467137

[58] Simpkin A, Cochran E, Cameron F, Dattani M, de Bock M, Dunger DB, Forsander G, Guran T, Harris J, Isaac I, Insulin Receptor and the Kidney: Nephrocalcinosis in Patients with Recessive INSR Mutations. Nephron Physiol. 2014;128:55–61. 10.1159/000366225 PMC4369119 25358339

[59] Bamborschke D, Özdemir Ö, Kreutzer M, Motameny S, Thiele H, Kribs A, Dötsch J, Altmüller J, Nürnberg P, Cirak S. Ultra-rapid emergency genomic diagnosis of Donahue syndrome in a preterm infant within 17 hours. Am J Med Genet Part A. 2021;185:90–6. 10.1002/ajmg.a.61917 33048476

[60] Rosenberg AM, Haworth JC, Degroot GW, Trevenen CL, Rechler MM. A case of leprechaunism with severe hyperinsulinemia. Am J Dis Child (1960). 1980;134:170–5. 10.1001/archpedi.1980.02130140044014 6986080

[61] Jospe N, Kaplowitz PB, Furlanetto RW. Homozygous nonsense mutation in the insulin receptor gene of a patient with severe congenital insulin resistance: leprechaunism and the role of the insulin-like growth factor receptor. Clin Endocrinol. 1996;45:229–35. 10.1046/j.1365-2265.1996.d01-1548.x 8881457

[62] Galderisi A, Tamborlane W, Taylor SI, Attia N, Moretti C, Barbetti F. SGLT2i Improves Glycemic Control in Patients With Congenital Severe Insulin Resistance. Pediatrics 2022, 150. 10.1542/peds.2021-055671 35652305

[63] Watanabe T, Abe Y. Bartter-like syndrome in patients with molecular defects of the insulin receptor gene. Acta Diabetol. 2014;51:891–2. 10.1007/s00592-014-0606-y 24913309

[64] Grasso V, Colombo C, Favalli V, Galderisi A, Rabbone I, Gombos S, Bonora E, Massa O, Meschi F, Cerutti F, Six cases with severe insulin resistance (SIR) associated with mutations of insulin receptor: Is a Bartter-like syndrome a feature of congenital SIR? Acta Diabetol. 2013;50:951–7. 10.1007/s00592-013-0490-x 23824322

[65] Hovnik T, Bratanič N, Podkrajšek KT, Kovač J, Paro D, Podnar T, Bratina N, Battelino T. Severe progressive obstructive cardiomyopathy and renal tubular dysfunction in Donohue syndrome with decreased insulin receptor autophosphorylation due to a novel INSR mutation. Eur J Pediatrics. 2013;172:1125–9. 10.1007/s00431-012-1901-7 23229189

[66] Falik Zaccai TC, Kalfon L, Klar A, Elisha MB, Hurvitz H, Weingarten G, Chechik E, Fleisher Sheffer V, Haj Yahya R, Meidan G, Two novel mutations identified in familial cases with Donohue syndrome. Mol Genet Genom Med. 2014;2:64–72. 10.1002/mgg3.43 PMC3907912 24498630

[67] Melikyan MA, Ivannikova TE, Milovanova NV, Kolodkina AA, Bezlepkina OB. Mokrysheva: [Donohue syndrome and use of continuous subcutaneous IGF1 pump therapy]. Probl Endokrinol (Mosk). 2022;68:79–86. 10.14341/probl13121 PMC9762435 36337021

[68] Maassen JA, Klinkhamer MP, van der Zon GC, Sips H, Möller W, Krans HM, Lindhout D, Beemer FA. Fibroblasts from a leprechaun patient have defects in insulin binding and insulin receptor autophosphorylation. Diabetologia. 1988;31:612–7. 10.1007/BF00264769 2851471

[69] Desbois-Mouthon C, Danan C, Amselem S, Blivet-Van Eggelpoel MJ, Sert-Langeron C, Goossens M, Besmond C, Capeau J, Caron M. Severe resistance to insulin and insulin-like growth factor-I in cells from a patient with leprechaunism as a result of two mutations in the tyrosine kinase domain of the insulin receptor. Metab Clin Exp. 1996;45:1493–500. 10.1016/s0026-0495(96)90178-x 8969282

[70] Maassen JA, Klinkhamer MP, Odink RJ, Sips H, van der Zon GC, Wieringa T, Krans HM, Möller W. Improper expression of insulin receptors on fibroblasts from a leprechaun patient. Eur J Biochem. 1988;172:725–9. 10.1111/j.1432-1033.1988.tb13949.x 3280314

[71] Kirel B, Bozdağ Ö, Köşger P, Aydoğdu SD, Alıncak E, Tekin N. A case of Donohue syndrome Leprechaunism with a novel mutation in the insulin receptor gene. Turk pediatri arsivi. 2017;52:226–30. 10.5152/TurkPediatriArs.2017.3193 PMC5819861 29483803

[72] Almazán Monroy JD, Valle Martinez AJ, Milla Salguero SE, Perdomo Domínguez ES. Donohue syndrome with a homozygous INSR exon 14 deletion: a case report. Oxford medical case reports 2025, 2025:omaf183. 10.1093/omcr/omaf183 PMC12476552 41025026

[73] Mariani S, Pedone A, Meschi F, di Natale B, Caputo R, Broggi U, Chiumello G. Insulin resistance in a child with Acanthosis nigricans type A. Acta Paediatr Scand. 1982;71:667–70. 10.1111/j.1651-2227.1982.tb09496.x 7136687

[74] Kostopoulou E, Shah P, Ahmad N, Semple R, Hussain K. Gastrointestinal dysmotility and pancreatic insufficiency in 2 siblings with Donohue syndrome. Pediatr Diabetes. 2017;18:839–43. 10.1111/pedi.12483 28004474

[75] Azzabi O, Jilani H, Rejeb I, Siala N, Elaribi Y, Hizem S, Selmi I, Halioui S, Lascols O, Jemaa LB, Maherzi A. Arg924X homozygous mutation in insulin receptor gene in a Tunisian patient with Donohue syndrome. J Pediatr Endocrinol metabolism: JPEM. 2016;29:753–6. 10.1515/jpem-2015-0232 26974131

[76] Wang JL, Xu CZ, Wang JL, Liang XY, Wu W, Huang K, Dong GP. [A case of Rabson-Mendenhall syndrome treated with empagliflozin combined with metformin]. Zhonghua er ke za zhi = Chin J Pediatr. 2025;63:1144–6. 10.3760/cma.j.cn112140-20250319-00223 40962551

[77] Improda N, Katugampola H, Cerbone M, Shah P, Alexander S, Atterbury A, Hadj-Rabia S, Peters CJ, Semple RK, Dattani MT. Clinical spectrum of extreme insulin resistance syndromes treated with rhIGF-1: A single-center experience. J Clin Endocrinol Metab. 2026;111:2049–63. 10.1210/clinem/dgag031 PMC13271778 41606799

[78] Carmody D, Ladsaria SS, Buikema RK, Semple RK, Greeley SAW. Successful rhIGF1 treatment for over 5 years in a patient with severe insulin resistance due to homozygous insulin receptor mutation. Diabet medicine: J Br Diabet Association. 2016;33:e8–12. 10.1111/dme.12884 PMC4751063 26262567

[79] Plamper M, Gohlke B, Schreiner F, Woelfle J. Mecasermin in Insulin Receptor-Related Severe Insulin Resistance Syndromes: Case Report and Review of the Literature. Int J Mol Sci 2018, 19. 10.3390/ijms19051268 PMC5983765 29695048

[80] Globa E, Zelinska N, Johnson MB, Flanagan SE, De Franco E. Neonatal and early-onset diabetes in Ukraine: Atypical features and mortality. Diabet medicine: J Br Diabet Association. 2023;40:e15013. 10.1111/dme.15013 PMC10946703 36398453

[81] Baqir ZS, Al-Lawati TT, Al Hussaini SO, Al-Sinani A, Al-Said K, Al-Rashdi I. A novel leprechaunism mutation, Cys807Arg, in an Arab infant: a rare cause of hypoglycaemia. Paediatrics Int child health. 2012;32:183–5. 10.1179/2046905512Y.0000000004 22824672

[82] Longo N, Langley SD, Griffin LD, Elsas LJ. Two mutations in the insulin receptor gene of a patient with leprechaunism: application to prenatal diagnosis. J Clin Endocrinol Metab. 1995;80:1496–501. 10.1210/jcem.80.5.7538143 7538143

[83] Foglino R, Barbetti F, Morotti E, Castorani V, Rigamonti A, Frontino G, Barera G, Bonfanti R. Case report: A case of Rabson-Mendenhall syndrome: long-term follow-up and therapeutic management with empagliflozin. Front Genet. 2024;15:1414451. 10.3389/fgene.2024.1414451 PMC11228259 38978877

[84] Masunaga Y, Kinjo K, Murai Y, Fujisawa Y, Saitsu H, Ogata T. Insulin resistant diabetes mellitus in a girl with mild Rabson-Mendenhall syndrome: efficacy of sodium glucose co-transporter 2 inhibitor. Diabetol Int. 2025;16:182–7. 10.1007/s13340-024-00773-y PMC11769922 39877435

[85] Almotawa F, Mushiba AM, Alqahtani N, Mashi A. Rabson-Mendenhall Syndrome Nearly Misdiagnosed as Type 1 Diabetes Mellitus: A Case Report. Cureus. 2025;17:e81190. 10.7759/cureus.81190 PMC11936689 40144769

[86] Chrzanowska J, Lachowska J, Weimann M, Zubkiewicz-Kucharska A, Fornalczyk K, Kilis-Pstrusińska K, Smigiel R. Case Report: The long-term effects of the empagliflozin therapy on glycemia and renal function in a patient with Rabson-Mendenhall syndrome caused by two heterozygous variants in INSR gene. Front Endocrinol. 2025;16:1552690. 10.3389/fendo.2025.1552690 PMC11999824 40241988

[87] Regan FM, Williams RM, McDonald A, Umpleby AM, Acerini CL, O'Rahilly S, Hovorka R, Semple RK, Dunger DB. Treatment with recombinant human insulin-like growth factor (rhIGF)-I/rhIGF binding protein-3 complex improves metabolic control in subjects with severe insulin resistance. J Clin Endocrinol Metab. 2010;95:2113–22. 10.1210/jc.2009-2088 20233784

[88] Longo N, Singh R, Elsas LJ. Decreased half-life of insulin-like growth factor I in Rabson-Mendenhall syndrome. J Inherit Metab Dis. 2001;24:546–50. 10.1023/a:1012411709972 11757582

[89] McDonald A, Williams RM, Regan FM, Semple RK, Dunger DB. IGF-I treatment of insulin resistance. Eur J Endocrinol. 2007;157(Suppl 1):S51–56. 10.1530/EJE-07-0271 17785698

[90] Takahashi Y, Kadowaki H, Momomura K, Fukushima Y, Orban T, Okai T, Taketani Y, Akanuma Y, Yazaki Y, Kadowaki T. A homozygous kinase-defective mutation in the insulin receptor gene in a patient with leprechaunism. Diabetologia. 1997;40:412–20. 10.1007/s001250050695 9112018

[91] Jo W, Sudo S, Nakamura A, Endo D, Konno Y, Ishizu K, Tajima T. Development of endometrial carcinoma in a patient with leprechaunism (donohue syndrome). Clin Pediatr endocrinology: case Rep Clin investigations : official J Japanese Soc Pediatr Endocrinol. 2013;22:33–8. 10.1297/cpe.22.33 PMC3756185 23990696

[92] Okawa MC, Cochran E, Lightbourne M, Brown RJ. Long-Term Effects of Metreleptin in Rabson-Mendenhall Syndrome on Glycemia, Growth, and Kidney Function. J Clin Endocrinol Metab. 2022;107:e1032–46. 10.1210/clinem/dgab782 PMC8852213 34718628

[93] Skarulis MC, Celi FS, Mueller E, Zemskova M, Malek R, Hugendubler L, Cochran C, Solomon J, Chen C, Gorden P. Thyroid hormone induced brown adipose tissue and amelioration of diabetes in a patient with extreme insulin resistance. J Clin Endocrinol Metab. 2010;95:256–62. 10.1210/jc.2009-0543 PMC2805496 19897683

[94] Kushchayeva YS, Startzell M, Cochran E, Auh S, Sekizkardes H, Soldin SJ, Kushchayev SV, Dieckmann W, Skarulis M, Abdul Sater Z, Thyroid Hormone Effects on Glucose Disposal in Patients With Insulin Receptor Mutations. J Clin Endocrinol Metab. 2020;105:e158–171. 10.1210/clinem/dgz079 PMC7093053 31588494

[95] Kraemer S. The fragile male. BMJ. 2000;321:1609–12. 10.1136/bmj.321.7276.1609 PMC1119278 11124200

[96] van Westering-Kroon E, Huizing MJ, Villamor-Martinez E, Villamor E. Male Disadvantage in Oxidative Stress-Associated Complications of Prematurity: A Systematic Review, Meta-Analysis and Meta-Regression. Antioxid (Basel) 2021, 10. 10.3390/antiox10091490 PMC8465696 34573122

[97] Alur P. Sex Differences in Nutrition, Growth, and Metabolism in Preterm Infants. Front Pediatr. 2019;7:22. 10.3389/fped.2019.00022 PMC6374621 30792973

[98] Torres MJ, Kew KA, Ryan TE, Pennington ER, Lin CT, Buddo KA, Fix AM, Smith CA, Gilliam LA, Karvinen S, 17beta-Estradiol Directly Lowers Mitochondrial Membrane Microviscosity and Improves Bioenergetic Function in Skeletal Muscle. Cell Metab. 2018;27:167–e179167. 10.1016/j.cmet.2017.10.003 PMC5762397 29103922

[99] Semple RK, Sleigh A, Murgatroyd PR, Adams CA, Bluck L, Jackson S, Vottero A, Kanabar D, Charlton-Menys V, Durrington P, Postreceptor insulin resistance contributes to human dyslipidemia and hepatic steatosis. J Clin Investig. 2009;119:315–22. 10.1172/JCI37432 PMC2631303 19164855

[100] Huang-Doran I, Tomlinson P, Payne F, Gast A, Sleigh A, Bottomley W, Harris J, Daly A, Rocha N, Rudge S, Insulin resistance uncoupled from dyslipidemia due to C-terminal PIK3R1 mutations. JCI Insight. 2016;1:e88766. 10.1172/jci.insight.88766 PMC5070960 27766312

[101] McKinlay CJD, Alsweiler JM, Anstice NS, Burakevych N, Chakraborty A, Chase JG, Gamble GD, Harris DL, Jacobs RJ, Jiang Y, Association of Neonatal Glycemia With Neurodevelopmental Outcomes at 4.5 Years. JAMA Pediatr. 2017;171:972–83. 10.1001/jamapediatrics.2017.1579 PMC5710616 28783802

[102] Shah R, Dai DWT, Alsweiler JM, Brown GTL, Chase JG, Gamble GD, Harris DL, Keegan P, Nivins S, Wouldes TA, Association of Neonatal Hypoglycemia With Academic Performance in Mid-Childhood. JAMA. 2022;327:1158–70. 10.1001/jama.2022.0992 PMC8941348 35315886

[103] Wei X, Franke N, Alsweiler JM, Brown GTL, Gamble GD, McNeill A, Rogers J, Thompson B, Turuwhenua J, Wouldes TA, Neonatal Hypoglycemia and Neurocognitive Function at School Age: A Prospective Cohort Study. J Pediatr. 2024;272:114119. 10.1016/j.jpeds.2024.114119 PMC11688105 38815750

[104] Kleinridders A, Ferris HA, Cai W, Kahn CR. Insulin action in brain regulates systemic metabolism and brain function. Diabetes. 2014;63:2232–43. 10.2337/db14-0568 PMC4066341 24931034

[105] Faggioni R, Feingold KR, Grunfeld C. Leptin regulation of the immune response and the immunodeficiency of malnutrition. FASEB J. 2001;15:2565–71. 10.1096/fj.01-0431rev 11726531

[106] Saucillo DC, Gerriets VA, Sheng J, Rathmell JC, Maciver NJ. Leptin metabolically licenses T cells for activation to link nutrition and immunity. J Immunol. 2014;192:136–44. 10.4049/jimmunol.1301158 PMC3872216 24273001

[107] Tsai S, Clemente-Casares X, Zhou AC, Lei H, Ahn JJ, Chan YT, Choi O, Luck H, Woo M, Dunn SE, Insulin Receptor-Mediated Stimulation Boosts T Cell Immunity during Inflammation and Infection. Cell Metab. 2018;28:922–e934924. 10.1016/j.cmet.2018.08.003 30174303

[108] Fischer HJ, Sie C, Schumann E, Witte AK, Dressel R, van den Brandt J, Reichardt HM. The Insulin Receptor Plays a Critical Role in T Cell Function and Adaptive Immunity. J Immunol. 2017;198:1910–20. 10.4049/jimmunol.1601011 28115529

[109] Huang-Doran I, Kinzer AB, Jimenez-Linan M, Thackray K, Harris J, Adams CL, de Kerdanet M, Stears A, O'Rahilly S, Savage DB, Ovarian Hyperandrogenism and Response to Gonadotropin-releasing Hormone Analogues in Primary Severe Insulin Resistance. J Clin Endocrinol Metab. 2021;106:2367–83. 10.1210/clinem/dgab275 PMC8277216 33901270

[110] Brown RJ, Joseph J, Cochran E, Gewert C, Semple R, Gorden P. Type B Insulin Resistance Masquerading as Ovarian Hyperthecosis. J Clin Endocrinol Metab. 2017;102:1789–91. 10.1210/jc.2016-3674 PMC5470776 27911591

[111] Baker A, Kerkar N, Todorova L, Kamath BM, Houwen RHJ. Systematic review of progressive familial intrahepatic cholestasis. Clin Res Hepatol Gastroenterol. 2019;43:20–36. 10.1016/j.clinre.2018.07.010 30236549

[112] Zhou X, Sevilla-Gonzalez M, Phipps AI, Udler M, Castellvi-Bel S, Chan AT, Pellatt AJ, Schoen RE, Giovannucci E, Gunter MJ, Diabetogenic processes for insulin resistance-linked hyperinsulinaemia are associated with colorectal cancer. Diabetologia. 2026;69:942–52. 10.1007/s00125-025-06631-z PMC12957015 41398395

[113] Tsugane S, Inoue M. Insulin resistance and cancer: epidemiological evidence. Cancer Sci. 2010;101:1073–9. 10.1111/j.1349-7006.2010.01521.x PMC11159937 20345478

[114] Bruning JC, Michael MD, Winnay JN, Hayashi T, Horsch D, Accili D, Goodyear LJ, Kahn CR. A muscle-specific insulin receptor knockout exhibits features of the metabolic syndrome of NIDDM without altering glucose tolerance. Mol Cell. 1998;2:559–69. 10.1016/s1097-2765(00)80155-0 9844629

[115] Belke DD, Betuing S, Tuttle MJ, Graveleau C, Young ME, Pham M, Zhang D, Cooksey RC, McClain DA, Litwin SE, Insulin signaling coordinately regulates cardiac size, metabolism, and contractile protein isoform expression. J Clin Invest. 2002;109:629–39. 10.1172/JCI13946 PMC150890 11877471

[116] Bluher M, Michael MD, Peroni OD, Ueki K, Carter N, Kahn BB, Kahn CR. Adipose tissue selective insulin receptor knockout protects against obesity and obesity-related glucose intolerance. Dev Cell. 2002;3:25–38. 10.1016/s1534-5807(02)00199-5 12110165

[117] Tiwari S, Singh RS, Li L, Tsukerman S, Godbole M, Pandey G, Ecelbarger CM. Deletion of the insulin receptor in the proximal tubule promotes hyperglycemia. J Am Soc Nephrol. 2013;24:1209–14. 10.1681/ASN.2012060628 PMC3736710 23723425

